# Supplementary material for: Inactive disease in patients with lupus is linked to autoantibodies to type I interferons that normalize blood IFNα and B cell subsets
Source: Cell Rep Med. 2023 Jan 17;4(1):100894. doi: 10.1016/j.xcrm.2022.100894 (PMC9873953; doi:10.1016/j.xcrm.2022.100894)
Supplement: Document S1. Figures S1–S5 and Tables S1–S3 [file mmc1.pdf]

Cell Reports Medicine, Volume *4*

Supplemental information

Inactive disease in patients with lupus is linked to autoantibodies to type I interferons that normalize blood IFNα and B cell subsets

Hannah F. Bradford, Liis Haljasmägi, Madhvi Menon, Thomas C.R. McDonnell, Karita Särekannu, Martti Vanker, Pärt Peterson, Chris Wincup, Rym Abida, Raquel Fernandez Gonzalez, Vincent Bondet, Darragh Duffy, David A. Isenberg, Kai Kisand, and Claudia Mauri

Supplementary Figures

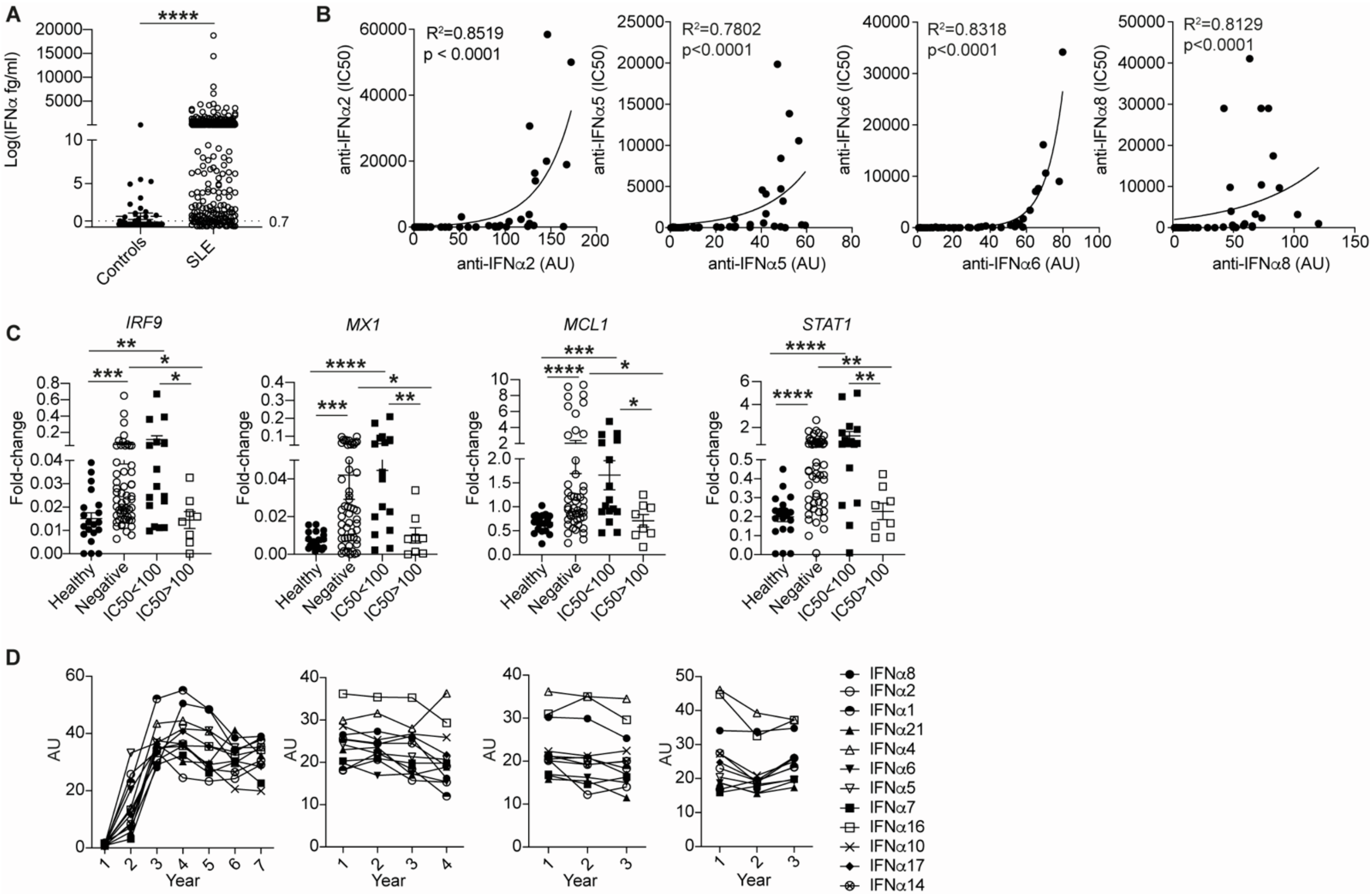

Supplementary Figure 1. High titres of anti-IFNα-autoantibodies are associated with high IFNα neutralizing capacity, reduced ISG expression and are specific to all IFNα subtypes tested (Related to Figure 1).

(A) Serum IFNα levels (fg/ml) for 54 healthy controls and 470 SLE patients.

(B) Correlations between anti-IFNα-autoantibody titres and neutralizing capacity (IC50) for autoantibody subtypes IFNα2, IFNα5, IFNα6 and IFNα8.

(C) Fold-change expression of individual ISGs *IRF9*, *MX1*, *MCL1* and *STAT1* for PBMCs isolated from SLE patients with neutralizing (n=8) or non-neutralizing anti-IFNα-autoantibodies (n=16), anti-IFNα-autoantibody negative SLE patients (n=54) and healthy controls (n=17), as measured by RT-qPCR relative to *GAPDH*.

(D) Titres of neutralizing anti-IFNα-autoantibodies against IFNα subtypes longitudinally in 4 SLE patients. *P<0.05, **P<0.01, ***P<0.001, ****P<0.0001 by unpaired Student's t-test with Welch's correction (A), two-tailed nonparametric Spearman correlation (B), Mann-Whitney test (C) or non-parametric Kruskal-Wallis test with Dunn's multiple comparison (D). Error bars are shown as mean±SEM.

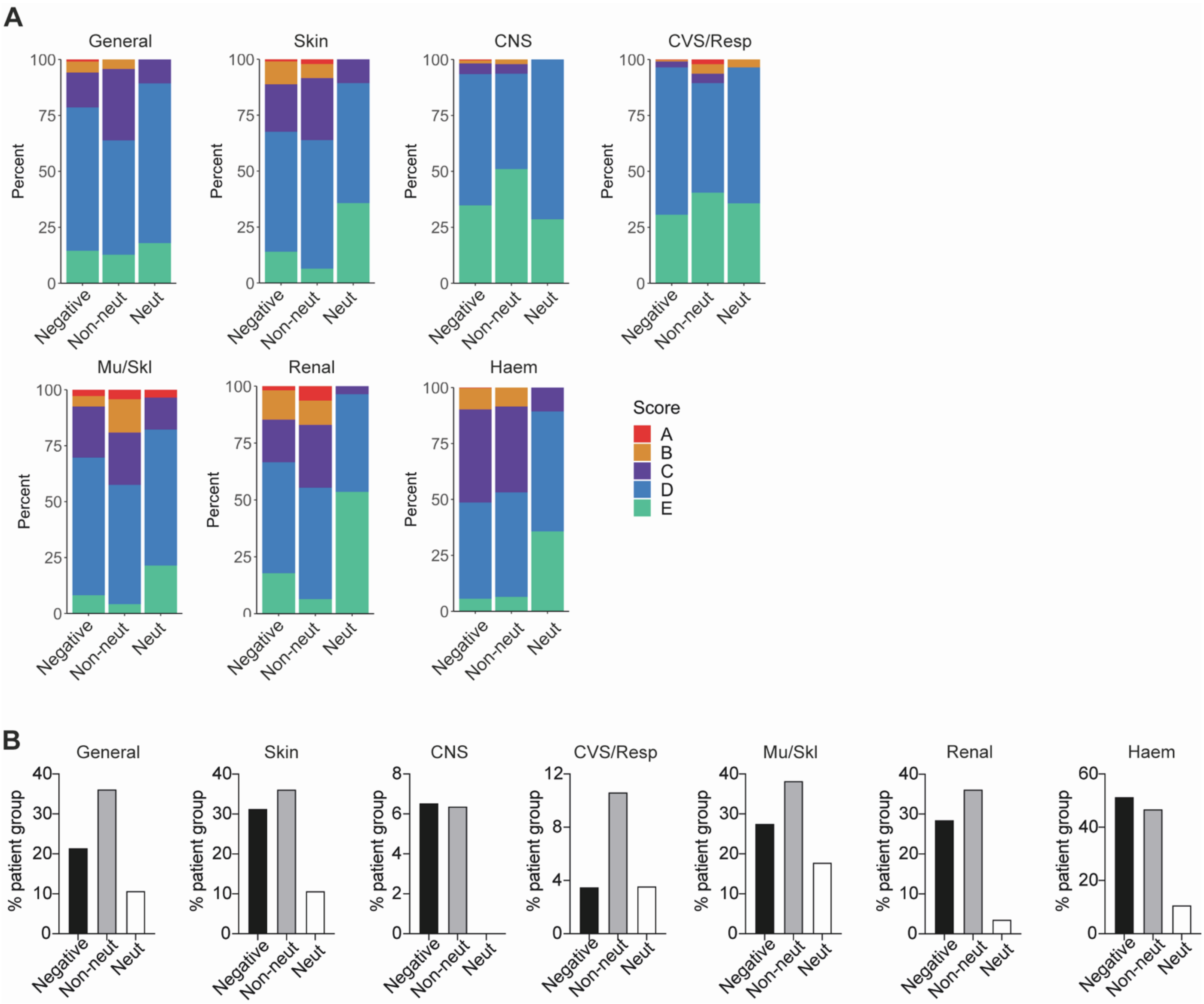

Supplementary Figure 2. Organ system involvement of SLE patients with neutralizing or non-neutralizing anti-IFNα-autoantibodies, and SLE patients lacking anti-IFNα-autoantibodies (Related to Figure 2).

(A) Percentages of SLE patients with neutralizing anti-IFNα-autoantibodies, non-neutralizing anti-IFNα-autoantibodies or anti-IFNα-autoantibody negative patients with A, B, C, D or E BILAG scores within each organ system.

(B) Percentages of SLE patients with high disease activity (a BILAG score of A, B or C) in individual organ systems, within patients with neutralizing or non-neutralizing anti-IFNα-autoantibodies, or anti-IFNα-autoantibody negative patients.

Abbreviations: central nervous system (CNS), cardiovascular/respiratory (CVS/Resp), musculoskeletal (Mu/Skl), haematological (Haem).

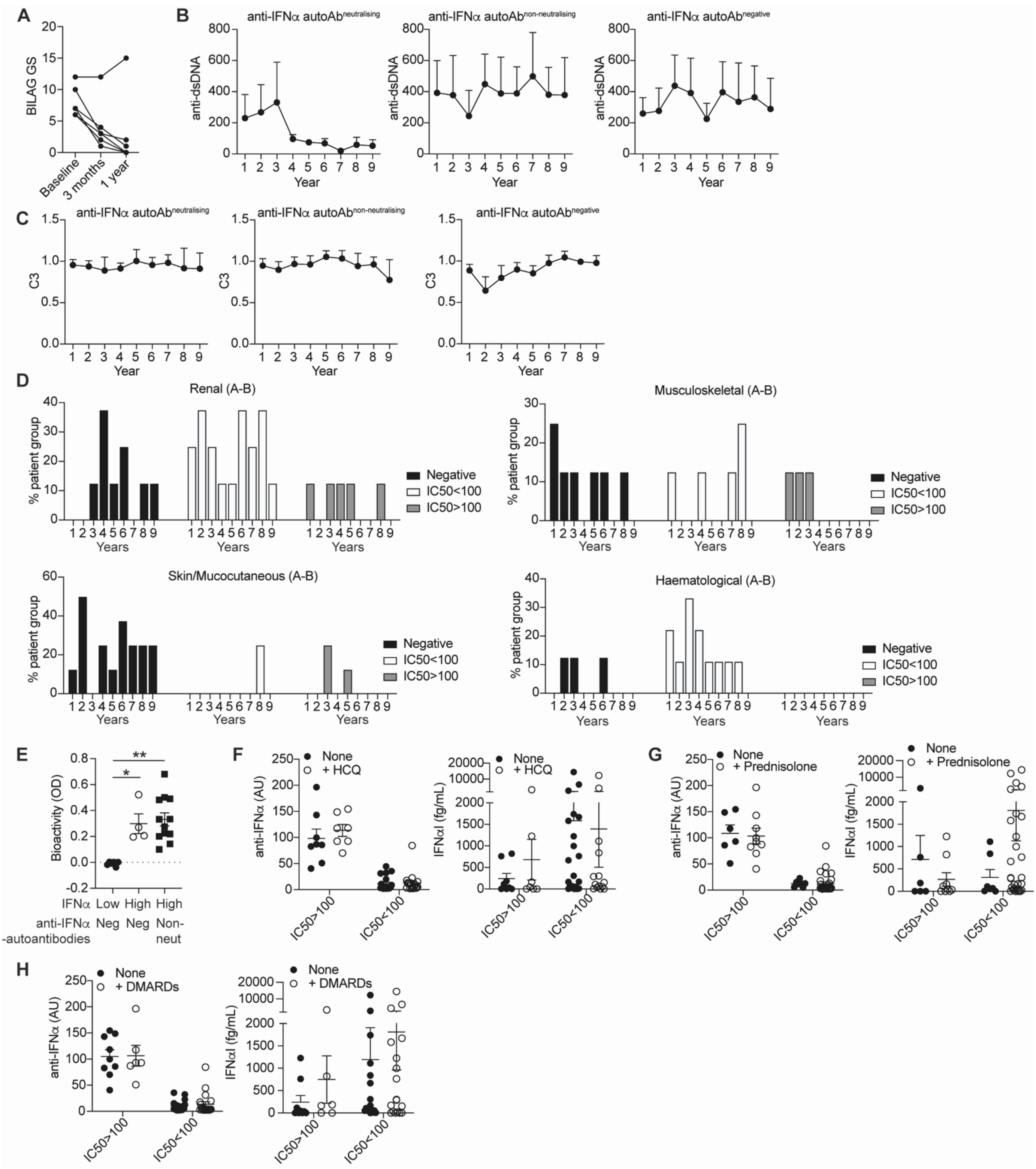

Supplementary Figure 3. Longitudinal association of clinical laboratory parameters and organ involvement for patients with neutralizing, non-neutralizing or negative anti-IFNα-autoantibodies, and cross-sectional association of treatment regime with anti-IFNα-autoantibody titres and serum IFNα levels (Related to Figure 2).

(A) Graph shows BILAG global scores (GS) for 6 patients with high disease activity at the time of initial sampling (baseline) and at 4 subsequent time points over a period of 3 years.

(B-C) Longitudinal analysis of serum (B) anti-dsDNA autoantibody titres and (C) C3 levels for SLE patients with neutralizing anti-IFNα-autoantibodies (n=11), non-neutralizing anti-IFNα-autoantibodies (n=10) and anti-IFNα-autoantibody negative patients (n=9).

(D) Graphs show percentages of patient groups with high disease activity (BILAG score of A or B) in renal, musculoskeletal, skin and haematological organ systems, for patients studied longitudinally with neutralizing anti-IFNα-autoantibodies (n=11), non-neutralizing anti-IFNα-autoantibodies (n=10) or anti-IFNα-autoantibody negative patients (n=9).

(E) Graph shows the bioactivity (OD) of IFNα in the sera from anti-IFNα-autoantibody negative patients with low or high levels of circulating IFNα, and from patients with non-neutralizing anti-IFNα-autoantibodies with high levels of circulating IFNα.

(F-H) Graphs show anti-IFNα-autoantibody titres and serum IFNα concentrations for SLE patients with neutralizing (IC50>100) versus non-neutralizing (IC50<100) anti-IFNα-autoantibodies who are receiving (F) hydroxychloroquine (HCQ), (G) prednisolone or (H) disease-modifying anti-rheumatic drugs (DMARDs, including methotrexate, mycophenolate mofetil and azathioprine).

*P<0.05, **P<0.01 by Kruskal-Wallis test with Dunn's multiple comparison. Error bars are shown as mean±SEM.

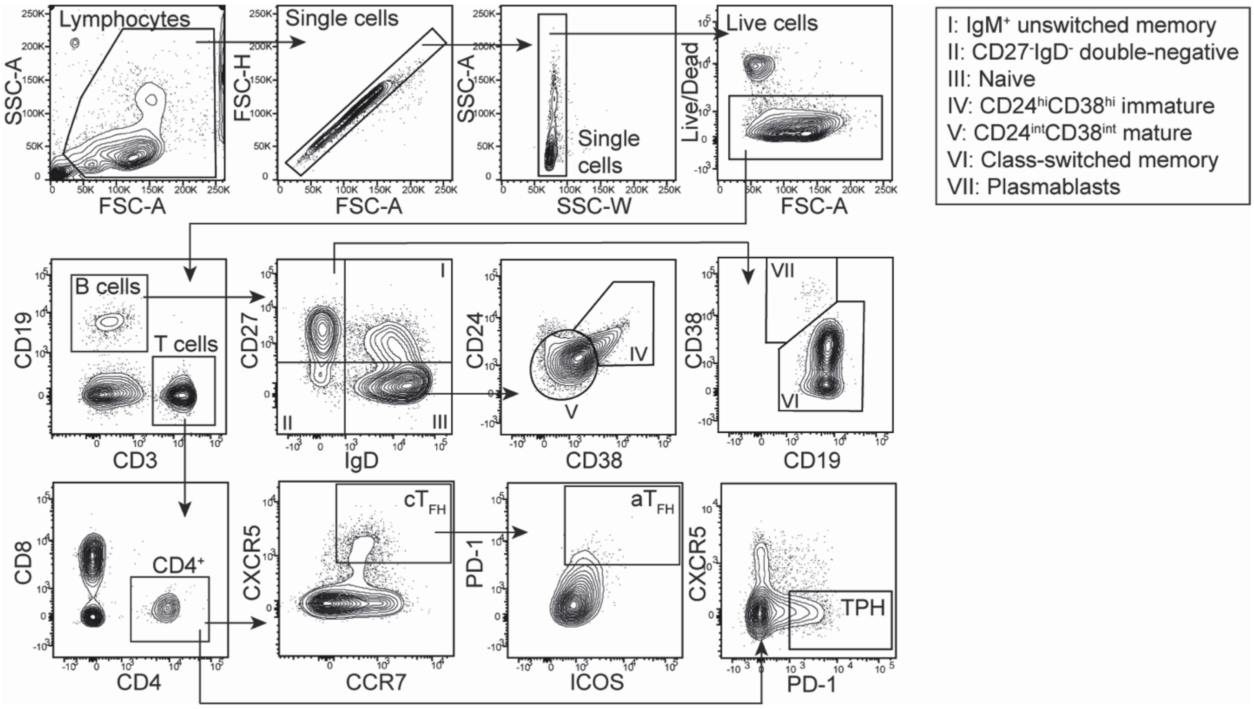

Supplementary Figure 4. Gating strategy for *ex vivo* measurement of B cell subset and T_{FH}/TPH subset frequencies by flow cytometry (Related to Figure 3 and Supplementary Figure 5).

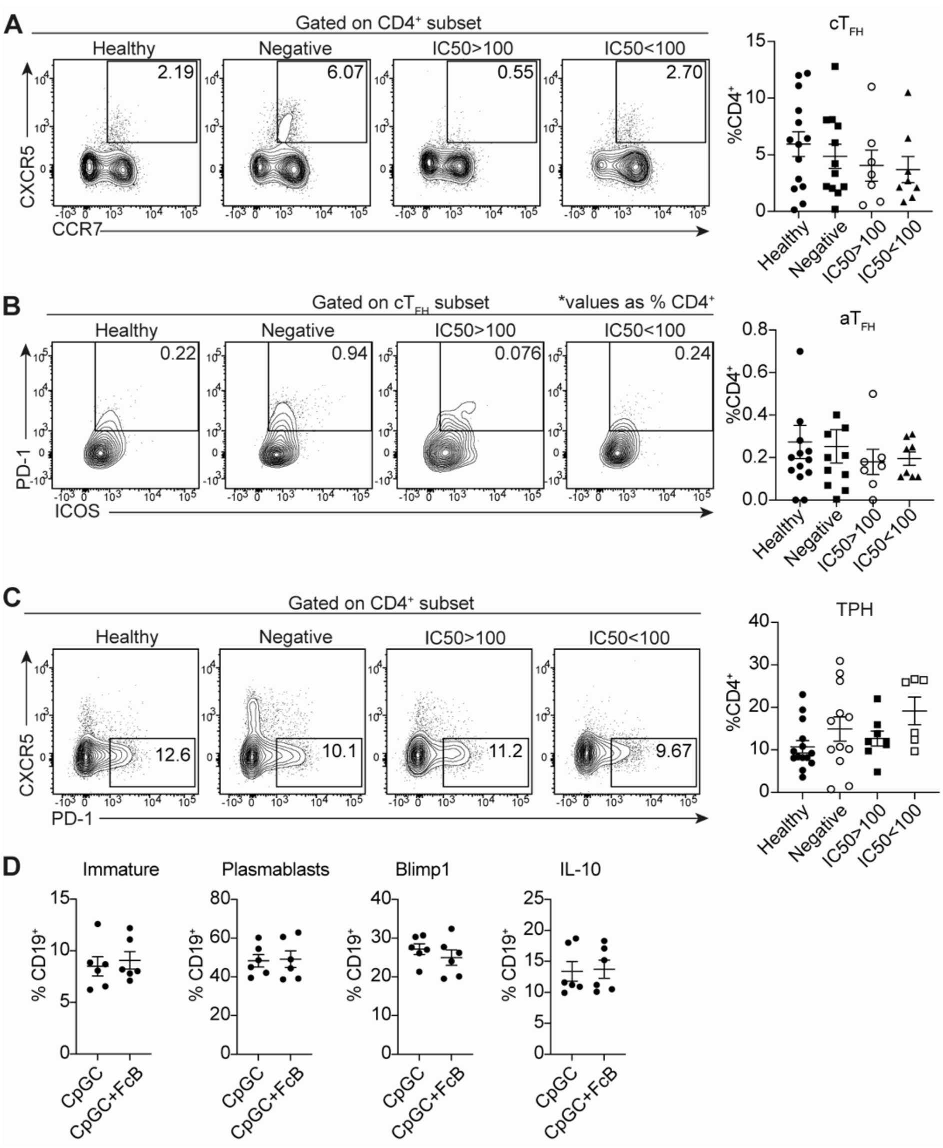

Supplementary Figure 5. Frequencies of circulating T follicular and T peripheral helper cells in SLE patients with neutralizing, non-neutralizing and negative anti-IFNα-autoantibodies and in healthy controls (Related to Figure 3).

(A-C) Representative contour plots and graphs show *ex vivo* frequencies of (A) classical T_{FH} (cT_{FH}), (B) activated T_{FH} (aT_{FH}) T cells and (C) T peripheral helper cells (TPH) in SLE patients with neutralizing anti-IFNα-autoantibodies (n=7), non-neutralizing anti-IFNα-autoantibodies (n=8), anti-IFNα-autoantibody negative patients (n=12 for cT_{FH}, n=11 for aT_{FH}) and healthy donors (n=14). Values are given as % total $CD4^+$ population.

(D) Graphs show frequencies of immature B cells, plasmablasts, $Blimp1^+$ and $IL\text{-}10^+$ B cells within the $CD19^+$ population following 72h stimulation of PBMCs with CpGC with and without Fc blocking reagent (FcB).

Supplementary Table 1. Prevalence of anti-cytokine autoantibodies in SLE patients and healthy controls (Related to Figure 1).

Seroactivity	Patients (%,n) (n=474)	Controls (%,n) (n=312)	Fisher's exact test:
IFN-α pool	14 (66)	2.2 (7)	P < 0.0001
IFN-ω	12 (59)	1.0 (3)	P < 0.0001
IFN-λ pool	2 (8)	1.0 (3)	P = 0.5404
IFN-β	1 (5)	0 (0)	P = 0.1631
IFN-γ	4 (18)	1.9 (6)	P = 0.2025
Th17 pool	6 (28)	1.6 (5)	P = 0.0031
IL pool	6 (28)	0.3 (1)	P < 0.0001
TNF pool	7 (35)	0.3 (1)	P < 0.0001
IL-1 pool	1 (4)	2.9 (9)	P = 0.0421
None	47 (223)	88.8 (277)	P < 0.0001

474 SLE patients and 312 healthy controls tested for autoantibodies against various cytokines with the Luciferase Immunoprecipitation system (LIPS) assay. The autoantibodies to cytokines were measured in groups that included an IFNα pool (IFNα1, IFNα2, IFNα8, IFNα21), IFNω, IFNγ, IFNβ1, a T helper (Th)17 pool, (IL-17A, IL-17F, IL-22), an IFNλ pool (IL-28A, IL-28B, IL-29), an interleukin (IL) pool (IL-6, IL-7, IL-10, IL-15), and a tumor necrosis factor (TNF) pool (TNF, LTA, BAFF, APRIL).

Supplementary Table 2. Demographic characteristics of SLE patients analysed longitudinally (Related to Figure 2).

Anti-IFNa autoAb	Neutralizing (IC50>100) (n=11)	Non-neutralizing (IC50<100) (n=10)	Negative (n=9)
Average age at start (years)	40.9 (24-67)	37.5 (17-70)	41.4 (21-61)
Average disease duration at start (years)	12.5 (3-25)	8.74 (0.6-20.0)	7.4 (1-15.2)
Ethnicity C/AC/SA/EA (%)	45.5/36.4/9.1/9.1	30.0/50.0/10.0/10.0	44.4/33.3/0.0/22.2
Gender F/M (%)	87.5/12.5	90.0/10.0	100.0/0.0

Abbreviations: Caucasian (C), Afro-Caribbean (A/C), South Asian (SA), East Asian (EA), Female (F), Male (M).

Supplementary Table 3. Demographics of SLE patients and healthy controls used for *ex vivo* B cell phenotyping. (Related to Figure 3).

	Healthy (n=15)	Negative (n=40)	Non-neutralizing (n=13)	Neutralizing (n=10)
Age (range)	38.2 (24-60)	47.38 (26-83)	43.54 (30-75)	45.6 (29-67)
Gender (%F:M)	86.7:13.3	97.5:2.5	84.6:23.1	100:0
Ethnicity % (C/AC/SA/EA)	53.3/13.3/13.3 /20	35/42.5/10/12.5	30.8/38.5/7.7/23.1	30/40/10/10
GS (Avg)	-	4.25	5.6	1.9
dsDNA (Avg)	-	91.4	103.1	70.1
C3 (Avg)	-	1	0.97	1.06
Organ involvement % (A/B/C/D/E)				
General	-	0/0/10/75/15	0/0/7.7/84.6/7.7	0/0/10/90/0
Skin	-	0/5/22.5/60/12.5	0/7.7/15.4/76.9/0	0/0/20/80/0
CNS	-	0/0/5/55/40	0/0/7.7/84.6/7.7	0/0/0/100/0
CVS/Resp	-	0/2.5/0/62.5/32.5	0/0/0/84.6/15.4	0/0/0/100/0
Mu/Skl	-	0/7.5/5/70/17.5	7.7/0/7.7/84.6/0	0/0/0/20/80
Renal	-	0/20/15/50/15	7.7/23.1/30.8/38.5/0	0/10/30/60/0
Haem	-	0/2.5/35/52.5/10	0/0/53.8/46.2/0	0/0/70/30/0

Abbreviations: Caucasian (C), Afro/Caribbean (A/C), South Asian (SA), East Asian (EA), global score (GS), double-stranded DNA (dsDNA), central nervous system (CNS), cardiovascular/respiratory (CVS/Resp), musculoskeletal (Mu/Skl), haematological (Haem).
