# Supplementary material for: Inactive disease in patients with lupus is linked to autoantibodies to type I interferons that normalize blood IFNα and B cell subsets
Source: Cell Rep Med. 2023 Jan 17;4(1):100894. doi: 10.1016/j.xcrm.2022.100894 (PMC9873953; doi:10.1016/j.xcrm.2022.100894)
Supplement: Document S2. Article plus supplemental information [file mmc2.pdf]

Cell Reports
Medicine

Report

Inactive disease in patients with lupus is linked to autoantibodies to type I interferons that normalize blood IFNα and B cell subsets

Graphical abstract

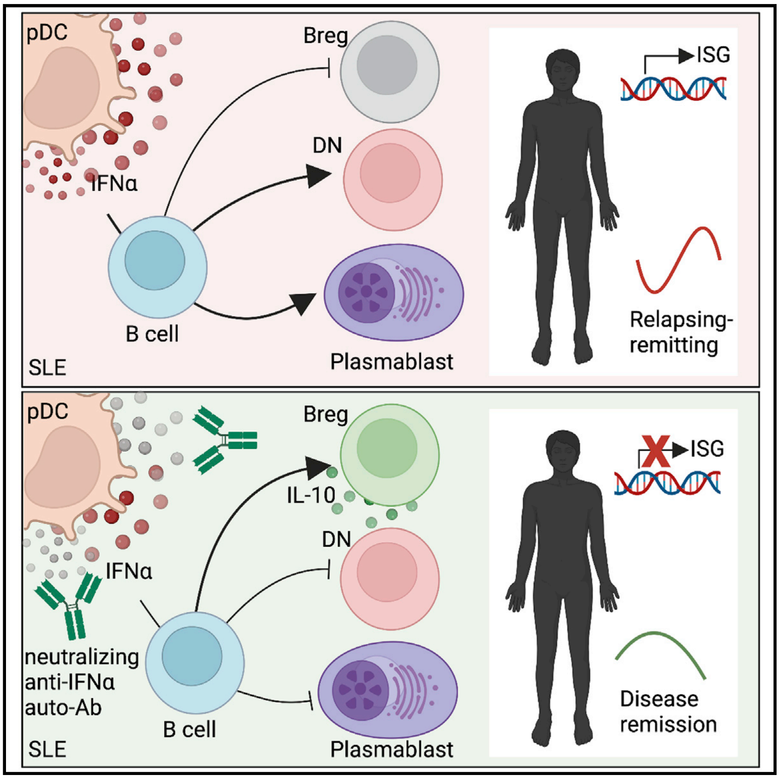

Highlights

- 14% of patients with SLE harbor natural anti-IFNα-Abs
- Neutralizing anti-IFNα-Abs are associated with reduced serum IFNα levels
- Neutralizing anti-IFNα-Abs are associated with reduced disease activity
- Normalization of B cell subsets in patients with SLE with neutralizing anti-IFNα-Abs

Authors

Hannah F. Bradford, Liis Haljasmägi, Madhvi Menon, ..., David A. Isenberg, Kai Kisand, Claudia Mauri

Correspondence

hannah.bradford.12@ucl.ac.uk (H.F.B.),
madhvi.menon@manchester.ac.uk (M.M.),
kai.kisand@ut.ee (K.K.),
c.mauri@ucl.ac.uk (C.M.)

In brief

Bradford et al. characterize the prevalence of anti-IFNα-Abs in patients with SLE and their association with serum levels of IFNα, clinical parameters, and B cell abnormalities. Patients with SLE harboring autoantibodies that neutralize IFNα show reduced serum IFNα levels and ISG expression, disease severity, and normalized B cell compartments.

Bradford et al., 2023, Cell Reports Medicine *4*, 100894
January 17, 2023 Crown Copyright © 2022
https://doi.org/10.1016/j.xcrm.2022.100894

CellPress

Cell Reports Medicine

CellPress
OPEN ACCESS

Report

Inactive disease in patients with lupus is linked to autoantibodies to type I interferons that normalize blood IFNα and B cell subsets

Hannah F. Bradford,[1,2,7,*] Liis Haljasmägi,[3,7] Madhvi Menon,[4,7,*] Thomas C.R. McDonnell,[5] Karita Särekannu,[3] Martti Vanker,[3] Pärt Peterson,[3] Chris Wincup,[2] Rym Abida,[2] Raquel Fernandez Gonzalez,[2] Vincent Bondet,[6] Darragh Duffy,[6] David A. Isenberg,[2] Kai Kisand,[3,8,*] and Claudia Mauri[1,2,8,9,*]

[1]Division of Infection and Immunity and Institute of Immunity and Transplantation, Royal Free Hospital, University College London, London NW3 2PP, UK
[2]Centre for Rheumatology, Division of Medicine, University College London, London WC1E 6JF, UK
[3]Institute of Biomedicine and Translational Medicine, University of Tartu, Tartu, Estonia
[4]Lydia Becker Institute of Immunology and Inflammation, Division of Infection, Immunity & Respiratory Medicine, School of Biological Sciences, University of Manchester, Manchester M13 9PL, UK
[5]Department of Biochemical Engineering, University College London, London WC1E 6BT, UK
[6]Translational Immunology Unit, Institut Pasteur, Université Paris Cité, Paris, France
[7]These authors contributed equally
[8]These authors contributed equally
[9]Lead contact
*Correspondence: hannah.bradford.12@ucl.ac.uk (H.F.B.), madhvi.menon@manchester.ac.uk (M.M.), kai.kisand@ut.ee (K.K.), c.mauri@ucl.ac.uk (C.M.)
https://doi.org/10.1016/j.xcrm.2022.100894

SUMMARY

Systemic lupus erythematosus (SLE) is characterized by increased expression of type I interferon (IFN)-regulated genes in 50%–75% of patients. We report that out of 501 patients with SLE analyzed, 73 (14%) present autoantibodies against IFNα (anti-IFN-Abs). The presence of neutralizing-anti-IFN-Abs in 4.2% of patients inversely correlates with low circulating IFNα protein levels, inhibition of IFN-I downstream gene signatures, and inactive global disease score. Hallmarks of SLE pathogenesis, including increased immature, double-negative plasmablast B cell populations and reduction in regulatory B cell (Breg) frequencies, were normalized in patients with neutralizing anti-IFN-Abs compared with other patient groups. Immunoglobulin G (IgG) purified from sera of patients with SLE with neutralizing anti-IFN-Abs impedes CpGC-driven IFNα-dependent differentiation of B cells into immature B cells and plasmablasts, thus recapitulating the neutralizing effect of anti-IFN-Abs on B cell differentiation *in vitro.* Our findings highlight a role for neutralizing anti-IFN-Abs in controlling SLE pathogenesis and support the use of IFN-targeting therapies in patients with SLE lacking neutralizing-anti-IFN-Abs.

INTRODUCTION

Systemic lupus erythematosus (SLE) is a heterogeneous autoimmune disease affecting multiple organ systems. Abnormal B cell proportions including expansion of atypical memory, also known as double-negative (DN) B cells, and autoantibody-secreting plasma cells contribute to autoimmune inflammation and tissue injury.[1–3] In addition to B cell dysfunction, ~50%–75% of patients with SLE present an upregulation of type I interferon (IFN-I)-stimulated genes (ISGs) that directly correlate with disease severity. The IFN-I family includes IFNβ, IFNω, IFNε, IFNκ, and 13 additional subtypes of IFNα that bind to the same receptor, IFNAR.[4] We and others have previously shown that a finely tuned IFNα response is required to induce the differentiation of immature B cells into plasma cells that produce antibodies during, for example, viral infection, as well as regulatory B cells (Bregs) that restore homeostasis.[5,6] In SLE, chronic IFNα production fuels autoimmunity by promoting the differentiation of monocytes to dendritic cells (DCs),[7,8] which activate autoreactive T cells; the generation of effector and memory $CD8^+$T cells[9–11]; and the differentiation of B cells into autoantibody-producing plasma cells but not Bregs.[5,12] The pathogenic role of IFNα in SLE is supported by several clinical observations. Patients with monogenic diseases, including complement and FASL deficiency and TREX-1 mutation, which all lead to IFN-I overproduction, display SLE-like symptoms.[13–15] Patients treated with IFN-I for cancer and chronic infections develop a lupus-like disease and/or anti-double-stranded DNA (dsDNA) antibodies.[16,17] IFN-α kinoid vaccination induces antibodies that cross-neutralize all IFNα subtypes, which in ~50% of immunized SLE patients has shown therapeutic efficacy.[18] IFN-I blockade has also been shown to be beneficial in patients with SLE.[19,20]

Crown Copyright © 2022
This is an open access article under the CC BY license (http://creativecommons.org/licenses/by/4.0/).

Table 1. Demographic and clinical characteristics of patients with SLE with neutralizing or non-neutralizing anti-IFN-Abs, anti-IFN-Ab-negative patients, and controls

	Control (n = 312)	Cross-sectional Ab^{neg} (n = 428)	Cross-sectional Ab$^{non-neut}$ (n = 47)	Cross-sectional Ab^{neut} (n = 28)
Age (range)	66.0 (31–87)	47.0 (17–86)	45.1 (23–73)	47.5 (28–72)
Age at diagnosis (range)	–	29.1 (1–75)	29.5 (12–63)	28.4 (8–51)
Gender (female:male)	(146:166)	(394:34)	(42:5)	(26:2)
Gender (% female:male)	(47.8:53.2)	(92:8)	(89.4:10.6)	(92.9:7.1)
Ethnicity (% AC/W/SA/EA/O)	(0/100/0/0/0)	(17.9/60.9/13.4/4.9/2.8)	(38.3/46.8/12.7/2.1/0)	(42.8/39.3/10.7/7.1/0)
Treatment (%)				
HCQ	–	49.4	35.6	39.3
Pred	–	50.1	68.9	42.9
MTX	–	3.8	6.7	0
MMF	–	9.6	22.2	14.3
Aza	–	16.2	17.8	4.8

Patients fulfilling the revised classification criteria for SLE were assessed for disease activity with the British Isles Lupus Assessment Group Index (BILAG). The BILAG index is a clinical measure of disease that distinguishes activity in nine different organ systems. Each organ system was given a grade, A, B, C, D, or E, where A was the most active and E the least active. Grades were converted into numerical scores using the BILAG-2004 index, where A = 12, B = 8, C = 1, D = 0, and E = 0. Global BILAG scores were calculated by adding the sum of the values from all organ systems. Patients with a global score higher than 6 were considered active. The following abbreviations are used: AC, African-Caribbean; W, White; SA, South Asian; EA, East Asian; O, other; HCQ, hydroxychloroquine; Pred, prednisolone; MTX, methotrexate; MMF, mycophenolate mofetil; Aza, azathioprine. Reported in this table are all patients measured for anti-IFN-Abs throughout the duration of the study.

Neutralizing autoantibodies to IFN-I has been reported to develop in patients treated with IFNα2 or IFNβ therapy[21,22]; in the majority of patients with autoimmune polyendocrinopathy syndrome type I (APS-1)[23,24] or thymoma[25]; at lower frequencies in rheumatic diseases, including cross-sectional lupus cohorts[26–28]; and more recently in a subset of patients with life-threatening COVID-19.[29,30]

Here, we showed that neutralizing autoantibodies against IFNα (anti-IFN-I-Abs) cross-react with all IFNα subtypes in a cross-sectional and longitudinal cohort of patients with SLE and are associated with significantly reduced levels of circulating IFNα levels, disease activity, and restored B cell responses, suggesting a disease-aggravating role for non-neutralizing anti-IFN-Abs.

RESULTS

Neutralizing anti-IFN-Abs reduce circulating IFNα and IFNα downstream signaling

To evaluate whether patients with SLE develop endogenous autoantibodies to IFNα or/and other cytokines, we tested sera from 474 patients with SLE and 312 healthy controls (controls) for autoantibodies (autoAbs) against cytokines with the luciferase immunoprecipitation system (LIPS) assay (clinical characteristic, genders, and ethnicities are reported in Table 1, and the study population described in detail in the STAR Methods). AutoAbs to cytokines were measured in groups that included IFNα (IFNα1, IFNα2, IFNα8, IFNα21); IFNω; IFNγ; IFNβ1; T helper 17 (Th17; IL-17A, IL-17F, IL-22); IFNλ (IL-28A, IL-28B, IL-29); interleukin (IL; IL-6, IL-7, IL-10, IL-15, IL-1β); and tumor necrosis factor (TNF; TNF, LTA, BAFF, APRIL) pools (Figure 1A; Table S2).

Most autoAbs to cytokines were either undetectable or produced at low concentrations in patient or control sera. High levels of autoAbs to IFNλ were detected in patients and controls (Figure 1A). We detected a significant increase in autoAbs to IFNα (66 out of 474 patients) and IFNω (59 out of 474 patients) in patients with SLE compared with controls (Figures 1B and 1C). Reactivity toward IFN-I subtypes was partially overlapping as 12% (n = 43) of patients had autoAbs to both IFNα and IFNω, whereas anti-IFNα or -IFNω single-positive patients comprised 4% each. Interestingly, the levels of anti-IFN-Abs significantly positively correlated with anti-IFNω-Abs (Figure 1D). Due to the well-established role of IFNα in promoting SLE pathogenesis, we focused our attention on the cohort of patients that displayed anti-IFN-Abs. Of note, anti-IFN-Abs were predominantly of the immunoglobulin G1 (IgG1) subclass (Figure 1E).

Quantification of serum IFNα levels with the ultrasensitive Simoa method[31] showed that 93% of patients with SLE had IFNα serum levels over the detection limit (0.7 fg/mL) compared with 30% of controls (Figure S1A). The presence of high titers of anti-IFN-Abs mirrored a significant reduction in the levels of circulating IFNα compared with those who were anti-IFN-Ab negative and with those with low anti-IFN-Ab titers (Figure 1F).

The capacity of anti-IFN-Abs to neutralize IFNα was assessed using a reporter-cell-line-based neutralization assay as previously described.[32] Serum samples with high anti-IFN-Ab levels were more efficient in blocking all tested subtypes (IFNα2, -5, -6, and -8) of IFNα bioactivity *in vitro* (Figures 1G and S1B). Only anti-IFN-Abs with a neutralizing capacity of IC50 >100 negatively correlated with serum levels of IFNα (Figure 1H).

To gain mechanistic insight into the capacity of neutralizing anti-IFN-Abs to reduce downstream IFN-I signaling, we compared the IFN-I composite score,[33] a cumulative measure of mRNA expression of four individual ISGs, *MX1*, *MCL1*, *IRF9*, and *STAT1* (see STAR Methods), in patients with SLE with and without anti-IFN-Abs and controls. IFN-I score was significantly

CellPress
OPEN ACCESS

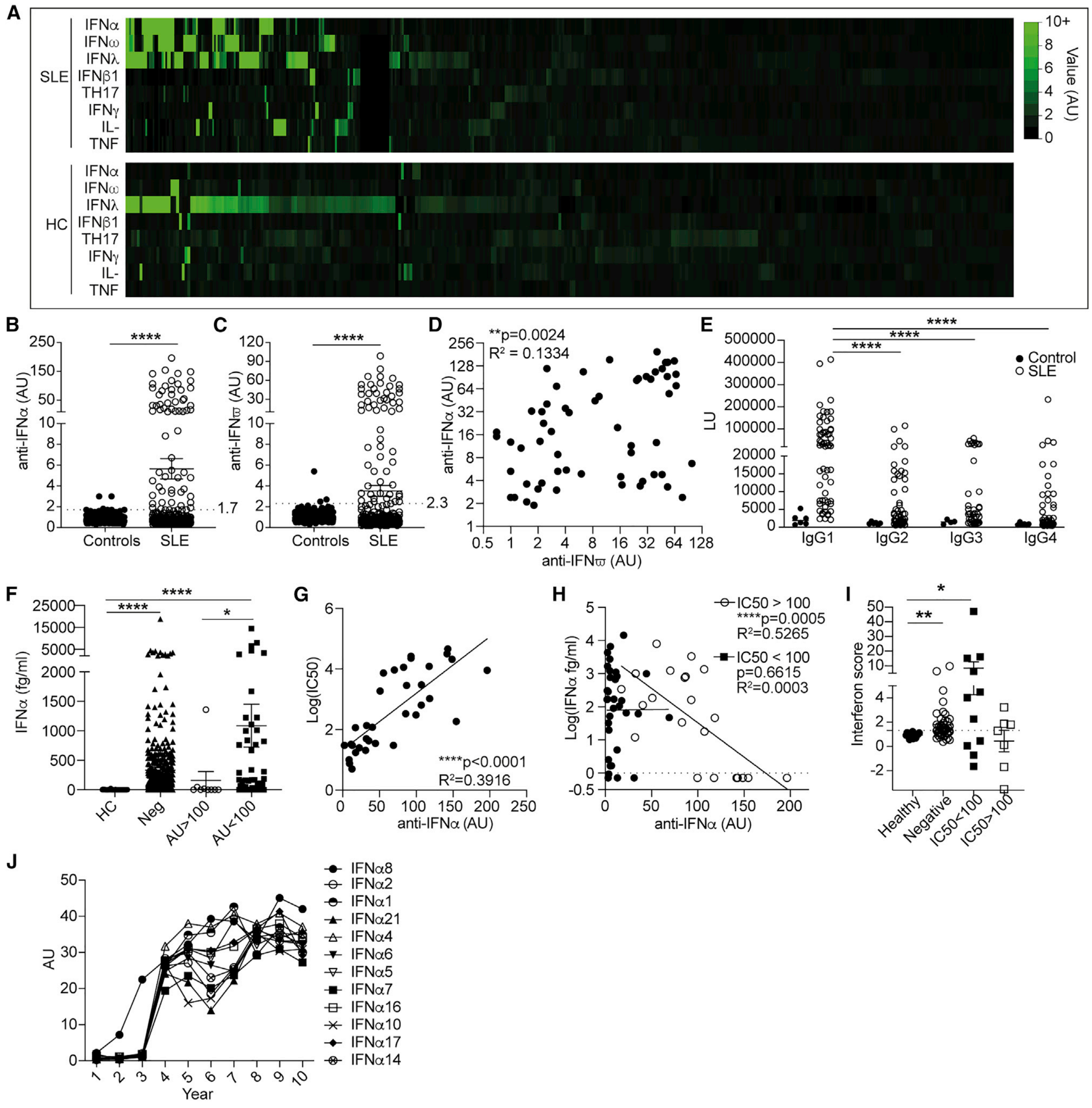

Figure 1. Neutralizing anti-IFN-Abs in patients with SLE inversely correlate with circulating IFNα

(A) Heatmap showing levels (arbitrary units [a.u.]) of anti-cytokine autoantibodies (IFNα pool, IFNω, IFNλ pool, IFNβ1, Th17 pool, IFNγ, IL-pool, and TNF) in 474 patients with SLE and 312 controls measured by luciferase immunoprecipitation system (LIPS) assay.

(B and C) Levels of (B) anti-IFNα (positive cutoff 1.7 a.u.) and (C) anti-IFNω autoantibodies (positive cut-off 2.3 a.u.) in sera from 474 patients with SLE and 312 controls.

(D) Correlation between serum titers of anti-IFNα and anti-IFNω-autoantibodies in autoantibody-positive patients.

(E) IgG subclasses of anti-IFN-Abs represented as luciferase units (LUs) for patients with SLE (n = 59) and controls (n = 6).

(F) Serum IFNα concentration of patients with SLE with high (>100 a.u.), low (<100 a.u.), or negative (<1.9 a.u.) titers of anti-IFN-Abs and controls.

(G) Correlation between IC50 and titer of neutralizing anti-IFN-Abs.

(H) Correlation between serum IFNα concentrations (measured by Simoa assay) and anti-IFNα-autoAb titers for patients with SLE grouped according to neutralization capacity (neutralizing IC50 > 100, non-neutralizing IC50 < 100).

(legend continued on next page)

higher in anti-IFN-Ab-negative and non-neutralizing anti-IFN-Ab patients compared with controls and with patients with neutralizing anti-IFN-Abs. The latter displayed an IFN-I score comparable to controls (Figures 1I and S1C). We measured anti-IFN-Ab titers longitudinally over an average of 10 years from the first sample collection. All patients tested have autoAbs against 12 subtypes of IFNα (IFNα1, -2, -4, -5, -6, -7, -8, -10, -14, -16, -17, and -21) at high titers (Figures 1J and S1D).

Neutralizing anti-IFN-Abs are a proxy for persistent low levels of IFNα and are associated with a better clinical outcome

We next investigated the effect that the presence of neutralizing anti-IFN-Abs has on disease severity. Patients with at least one neutralizing Ab against an IFNα subtype displayed significantly lower disease activity (as measured by the British Isles Lupus Assessment Group [BILAG] global score [GS]) compared with patients without anti-IFN-Abs in circulation or patients with non-neutralizing anti-IFN-Abs (Figure 2A). Notably, 5 out of 6 patients with neutralizing anti-IFN-Abs that had active disease (GS $\geq$ 5) at the time of sampling displayed a consistently reduced GS in the follow-up clinic appointments, suggesting that the generation of neutralizing anti-IFN-Abs precedes amelioration of disease (Figure S3A). The analysis of organ involvement is depicted in Figure S2. Renal, skin, and musculoskeletal involvement was more common in patients with non-neutralizing Abs than in patients negative for these Abs.

To understand the stability of the anti-IFN-Abs, we assessed the kinetics of anti-IFN-Ab production, circulating IFNα levels, and disease activity in a longitudinal cohort (30 patients with SLE) over a 10-year period (cohort's demographics is presented in Table S2). The presence of high titers of neutralizing anti-IFN-Abs mirrored a reduction of serum pan-IFNα protein to undetectable levels. The prolonged presence of neutralizing anti-IFN-Abs together with a consistently low IFNα concentration also paralleled a persistent inactive clinical score (Figure 2B). Patients with non-neutralizing anti-IFN-Abs in circulation present with high levels of serum IFNα and a more severe disease activity (Figure 2C). We also observed reduced titers of anti-dsDNA autoAbs in patients with neutralizing anti-IFN-Abs but no changes in C3 levels between the different groups (Figures S3B and S3C).

Follow-up analysis of organ involvement showed that both the negative and non-neutralizing groups experienced more disease flares in the renal, musculoskeletal, skin, and hematological systems compared with patients with neutralizing anti-IFN-Abs (Figure S3D). One individual in the neutralizing anti-IFN-Ab group maintained a B score in renal activity; however, this patient had consistently high Ab titers and neutralizing capacity with undetectable serum IFNα for the entire duration and displayed inactive disease in all other organ systems.

The bioactivity of IFNα from the sera of non-neutralizing anti-IFN-Ab and anti-IFN-Ab-negative patients was similar, confirming that non-neutralizing anti-IFN-Abs do not neutralize circulating IFNα (Figure S3E). These results suggest that non-neutralizing anti-IFN-Abs may stabilize circulating IFNα levels as previously suggested for other cytokines.[34–36] Patients lacking anti-IFN-Abs present active disease over time (Figure 2D). Neither the titers of anti-IFN-Abs nor IFNα serum levels were affected by treatment regime (Figures S3F–S3H).

Restored B cell populations in patients with SLE with neutralizing anti-IFN-Abs

Patients with SLE are known to present with a variety of B cell abnormalities, including increased frequencies of immature, DN B cells and plasmablasts and a decrease in Bregs.[1–3] Previous work by us and others has demonstrated that the level of exposure to IFNα determines immature B cell fate.[5,6,37] Whereas exposure of immature B cells to low-moderate concentrations of IFNα simultaneously expand both Bregs and plasmablasts, high concentrations of IFNα (observed in patients with SLE) biases B cell differentiation toward pro-inflammatory plasmablasts and plasma cells.[6] To evaluate whether the presence of neutralizing anti-IFN-Abs is associated with a normalization of the B cell frequencies and their responses, we quantified *ex vivo* B cell subset frequencies in patient groups defined by the presence or absence of neutralizing and non-neutralizing anti-IFN-Abs and controls (Table S3). Anti-IFN-Ab-negative patients showed a significant increase in immature, DN ($CD27^-IgD^-$) and plasmablast($CD27^+IgD^-CD38^{hi}$) B cells and a reduced frequency of unswitched memory (USM; $CD27^+IgD^+$) and class-switched memory ($CD27^+IgD^-CD38^{low}$) B cells compared with controls (Figures 3A and 3B; gating strategy in Figure S4A).

Patients with neutralizing (IC50 > 100) anti-IFN-Abs have similar B cell subset frequencies to controls except for class-switched memory ($CD27^+IgD^-CD38^{lo}$) B cells. In contrast, patients with non-neutralizing (IC50 < 100) anti-IFN-Abs display the same degree of altered subset frequencies as anti-IFN-Ab-negative patients (Figure 3C). We show no differences in the frequencies of T follicular helper cell (T_{FH}) subsets (circulating [cT_{FH}] or activated [aT_{FH}]) between patients and controls (Figures S5A and S5B). No differences were detected in $CD4^+$ $CXCR5^-PD\text{-}1^+$ T peripheral helper cells (TPH) frequencies, previously described to be expanded in patients with SLE and to be drivers of disease activity[38] between controls and any group of patients with SLE (Figure S5C). This supports a direct role of anti-IFN-Abs in normalizing B cell subset frequencies rather than indirectly via modifications to the T_{FH} or TPH compartment.

To establish whether B cells from patients with SLE with anti-IFN-Abs have regained the capacity to differentiate into Bregs (hereafter defined as $IL\text{-}10^+$ B cells), we stimulated peripheral blood mononuclear cells (PBMCs) from patients with SLE and

(I) Interferon score of PBMCs isolated *ex vivo* from patients with neutralizing (n = 8) or non-neutralizing (n = 16) anti-IFN-Abs, anti-IFN-Ab-negative patients with SLE (n = 54), and controls (n = 17). Data represented are a cumulative score of the expression of ISGs *MX1*, *MCL1*, *IRF9*, and *STAT1* measured by qRT-PCR and relative to *GAPDH*.

(J) Representative graph showing the titers of neutralizing anti-IFN-Abs against IFNα subtypes longitudinally in one patient with SLE.

*p < 0.05, **p = 0.01, ****p < 0.0001 by (D) unpaired Student's t test with Welch's correction, (E and F) Kruskal-Wallis test with Dunn's multiple comparison, (G) two-tailed Spearman correlation, and (I) Mann-Whitney test. Error bars are shown as mean ± SEM.

CellPress
OPEN ACCESS

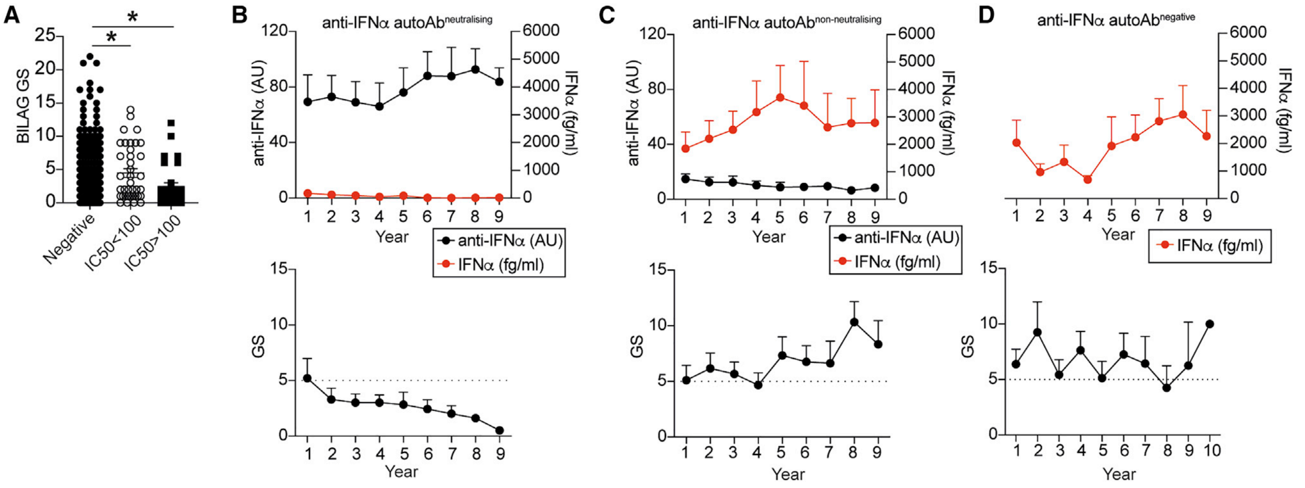

Figure 2. Neutralizing anti-IFN-Abs are longitudinally stable, neutralize IFNα *in vivo*, and are associated with inactive disease

(A) Graph shows disease activity as assessed by British Isles Lupus Assessment Group (BILAG) global score (GS) for patients with SLE with neutralizing (IC50 > 100) (n = 28) and non-neutralizing (IC50 < 100) anti-IFN-Abs (n = 47) and anti-IFN-Ab-negative patients (n = 375).

(B–D) Longitudinal analysis of anti-IFN-Ab titers, serum IFNα levels, and GSs for (B) 11 patients with SLE with neutralizing anti-IFN-Abs, (C) 10 patients with SLE with non-neutralizing anti-IFN-Abs, and (D) 9 anti-IFN-Ab-negative patients with SLE. Dotted lines at y = 5 indicate the point at which the GS is considered as active disease.

*p < 0.05 by (A) Kruskal-Wallis test with Dunn's multiple comparison. Error bars are shown as mean ± SEM.

controls with CpGC for 72 h to induce IFNα production by plasmacytoid DCs (pDCs) and IL-10$^+$ B cell differentiation, as previously shown by our group.[39] There was a significant decrease in IL-10$^+$ B cell frequencies in anti-IFNα-autoAb-negative and non-neutralizing anti-IFN-Ab patients but not in patients with neutralizing anti-IFN-Abs compared with controls (Figure 3D).

IFNα-induced immature and plasmablast B cell expansion is inhibited by IgG from patients with SLE with neutralizing anti-IFN-Abs

In response to viral infections, pDCs rapidly produce IFNα that drives B cell maturation into plasma cells producing Abs against viral antigens.[5] In view of the recent findings showing the detrimental effect of neutralizing anti-IFN-Abs in patients with COVID-19, it is important to understand the impact of neutralizing anti-IFN-Abs on "nascent" IFNα produced by challenged pDCs and how this affects healthy B cell differentiation. PBMCs from controls were stimulated with CpGC and cultured, respectively, with purified total IgG from patients with SLE with no Abs (negative), with non-neutralizing anti-IFN-Abs, and with neutralizing anti-IFN-Abs. Healthy allogeneic IgG was used as a control. An Fc blocking reagent was included to remove the IgG-mediated activation of FcR-expressing immune cell subsets. Inclusion of the Fc blocking reagent did not alter frequencies of immature B cells, plasmablasts, or Blimp1$^+$ or IL-10$^+$ B cells compared with CpGC stimulation alone (Figure S5D).

IgG from patients containing neutralizing anti-IFN-Abs significantly downregulated ISG expression in cultured CpGC-stimulated control PBMCs, confirming their ability to inhibit IFNα downstream signaling (Figure 4A). IgG from patients with neutralizing anti-IFN-Abs reduced the levels of IFNα in culture supernatants, whereas non-neutralizing anti-IFN-Abs increased IFNα concentrations (Figure 4B). Control IgG or IgG from patients with SLE lacking anti-IFN-Abs show no effect.

Addition of control IgG, or IgG from anti-IFN-Ab-negative patients with SLE, did not impair the CpG-induced expansion of immature B cells and plasmablasts. IgG isolated from patients with neutralizing anti-IFN-Abs significantly reduced the expansion of immature B cells and plasmablasts, with the latter also confirmed by a reduced Blimp1 expression, compared with non-neutralizing anti-IFN-Abs (Figures 4C and 4D). IgG from patients with non-neutralizing anti-IFN-Abs increased the frequencies of immature B cells and plasmablasts (and Blimp1$^+$ B cells), suggesting that these autoAbs stabilize IFNα and enhance B cell responses to IFNα. Only IgG from patients with neutralizing anti-IFN-Abs halted the CpGC-driven IL-10$^+$ B cell expansion, further confirming their neutralization capacity and the requirement of optimal IFNα levels for Breg differentiation (Figure 4E).

DISCUSSION

In summary, we report that a subset of patients with SLE harbor neutralizing anti-IFN-Abs that can modulate B cell responses and are associated with a better disease outcome. This is in contrast to patients with non-neutralizing low titers of anti-IFN-Abs, which appear to stabilize IFNα in the blood and expand circulating frequencies of DN memory B cells and plasmablasts. It has been previously shown that CD11c$^+$ DN B cells are pathogenic in SLE. Although we have not specifically measured this population, it is interesting that the DN B cells were reduced in patients with neutralizing anti-IFN-Abs. Future work with a larger cohort of patients quantifying frequencies of CD11c$^+$Tbet$^+$ DN B cells and their association with the development of neutralizing versus non-neutralizing anti-IFN-Abs are warranted.

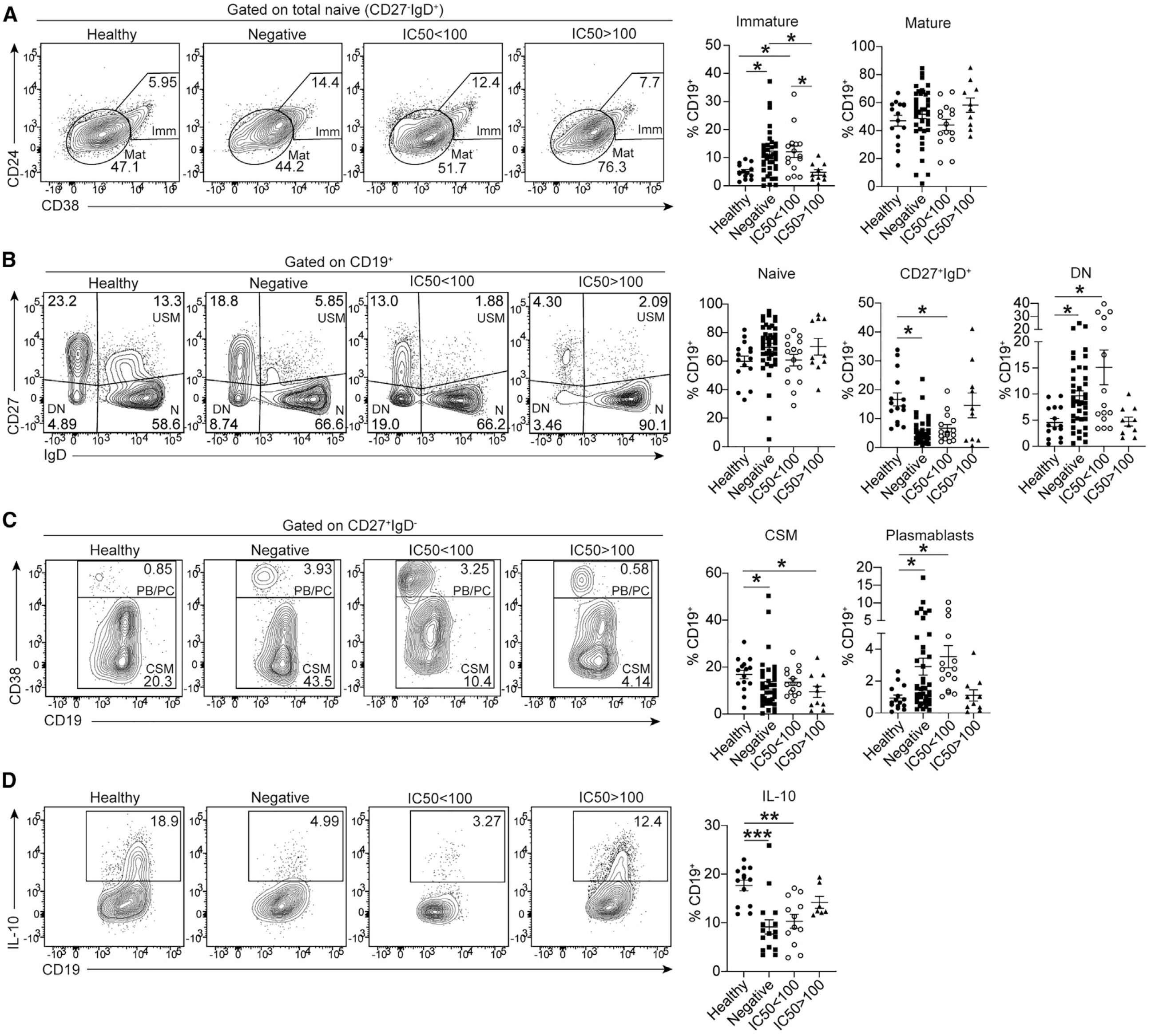

Figure 3. Patients with SLE with neutralizing anti-IFN-Abs display normalized frequencies of peripheral blood B cell subsets

(A–C) Representative contour plots and graphs shown for patients with SLE with neutralizing anti-IFN-Abs (n = 10) or non-neutralizing anti-IFN-Abs (n = 14), anti-IFN-Ab-negative patients with SLE (n = 41), and healthy individuals (n = 15). *Ex vivo* frequencies of (A) immature (Imm; $CD24^{hi}CD38^{hi}$) and mature (Mat; $CD24^{int}$ $CD38^{int}$) B cells gated within the naive ($CD27^{-}IgD^{+}$) subset; (B) naive (N; $CD27^{-}IgD^{+}$ IgM^{+}) unswitched memory (USM; $CD27^{+}$ IgD^{+}) and double-negative (DN; $CD27^{-}IgD^{-}$) B cells gated within the total $CD19^{+}$ population; and (C) class-switched memory B cells (CSMs) and plasmablasts/plasma cells (PB/PC) gated within the $CD27^{+}IgD^{-}$ subset. All values are given as the percentage of total $CD19^{+}$ population (gating strategy in Figure S3A).

(D) Representative contour plots and graphs show frequencies of IL-10^{+} B cells within the total $CD19^{+}$ population following 72 h *in vitro* CpGC stimulation of PBMCs isolated from patients with SLE with neutralizing anti-IFN-Abs (n = 7) or non-neutralizing anti-IFN-Abs (n = 12), anti-IFN-Ab-negative patients with SLE (n = 16), and healthy individuals (n = 13).

*$p < 0.05$, **$p < 0.01$, ***$p < 0.001$ by non-parametric Kruskal-Wallis test with Dunn's multiple comparison. Error bars are shown as mean ± SEM. Data are representative of at least 3 independent experiments.

The association of non-neutralizing anti-IFN-Abs with high IFNα concentrations is intriguing. It has been previously suggested that in certain cases, including more recently in patients with COVID-19,[40] circulating autoAbs can increase the half-life of the molecule they bind, possibly through the uptake and release of immune complexes by the neonatal Fc receptor on endothelial cells.[41,42] In addition, autoAb binding may change the conformation of IFNα and lead to more efficient binding to the receptor.

The cellular source of these anti-IFN-Abs remains unknown. It is plausible to speculate that anti-IFN-Abs could be produced either by a pool of memory B cells that, upon IFNα

CellPress
OPEN ACCESS

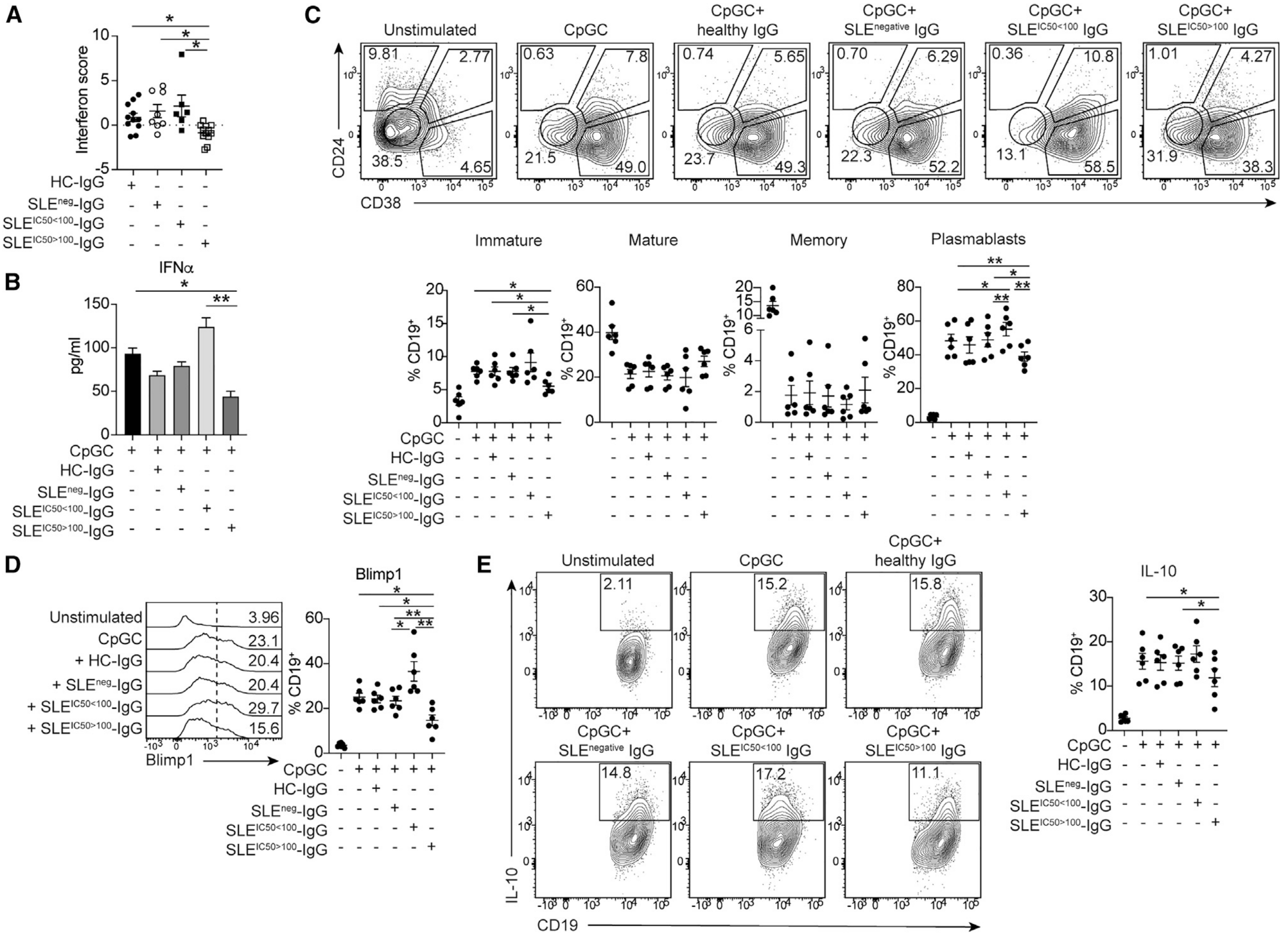

Figure 4. IgG from patients with neutralizing anti-IFN-Abs inhibits healthy B cell responses to IFNα PBMCs from controls were stimulated with CpGC in the presence of IgG purified from controls, patients with SLE lacking anti-IFN-Abs, or patients with SLE with non-neutralizing or neutralizing anti-IFN-Abs

(A) Graphs shows IFN score of control PBMCs following IgG exposure.

(B) Graph showing levels of IFNα in culture supernatants following IgG exposure.

(C) Representative contour plots and graphs show frequencies of $CD24^{hi}CD38^{hi}$ (Imm), $CD24^{int}CD38^{int}$ (Mat), $CD24^{+}CD38^{lo}$ (Memory [Mem]) B cells, and $CD24^{lo/-}CD38^{hi}$ (PBs).

(D) Histogram and graph show frequencies of $Blimp^{+}$ B cells following culture.

(E) Representative contour plots and graph show frequencies of IL-10^{+} B cells.

*$p < 0.05$, **$p < 0.01$ by one-way ANOVA with Tukey's multiple comparisons test (A and C–E) or Mann-Whitney test (B). Error bars are shown as mean ± SEM. Data are representative of 2 independent experiments.

challenge, such as following infection, induce the production of anti-IFN-Abs. However, our findings showing a persistent presence of autoAbs matching dramatically reduced levels of IFN-I and clinical score suggest a role for long-lived plasma cells in the production of these Abs. Due to the reduced disease severity afforded by the presence of high titers of neutralizing anti-IFN-Abs, none of these patients were treated with rituximab, which would abrogate circulating IFNα-specific memory B cells.

Our findings are relevant in the current COVID-19 pandemic, where anti-IFN-I-Abs and impaired IFN signaling have been associated with higher susceptibility for serious illness.[29] When administering anti-IFN-I blockade therapy (e.g., anifrolumab, a human monoclonal Ab to IFN-I subunit 1), measuring levels of anti-IFN-Abs in patient sera would be clinically more practical than measuring the IFN-I PBMC gene signature for pre-screening patients. Anifrolumab has been now approved as a therapy for patients with SLE with moderate and severe disease. It would be important to pre-screen patients to establish the presence and neutralization capacity of anti-IFN-Abs and exclude these patients from this treatment.

Limitations of the study

The scale of our analysis of B cells/PBMCs from these patients was limited by restricted sample availability due to the COVID-19 pandemic.

As discussed, the cellular source of neutralizing and non-neutralizing anti-IFN-Abs remains to be determined. Unfortunately,

this type of analysis requires a substantial amount of peripheral blood, which we are unable to obtain both because patients with SLE are frequently lymphopenic and our ethics only permit us to draw 25 mL blood per clinic visit.

The pathogenic role of non-neutralizing autoAbs through stabilization of IFNα levels in the circulation was suggested through indirect evidence; this has yet to be formally proven. Our study was also unable to discriminate autoAb avidity from concentration.

STAR★METHODS

Detailed methods are provided in the online version of this paper and include the following:

- KEY RESOURCES TABLE
- RESOURCE AVAILABILITY
  - Lead contact
  - Materials availability
  - Data and code availability
- EXPERIMENTAL MODEL AND SUBJECT DETAILS
  - Study population
  - Cell and cell lines
- METHOD DETAILS
  - PBMC and serum isolation
  - Luciferase immunoprecipitation system (LIPS) assay
  - Neutralization assay
  - IFNα concentration measurement
  - RT-qPCR
  - Flow Cytometry staining and analysis
  - IgG isolation from plasma
- QUANTIFICATION AND STATISTICAL ANALYSIS

SUPPLEMENTAL INFORMATION

Supplemental information can be found online at https://doi.org/10.1016/j.xcrm.2022.100894.

ACKNOWLEDGMENTS

This work is funded by a Versus Arthritis UK program grant (21140) to C.M.; the Versus Arthritis UK Research Award (21786) to C.M. and M.M.; the Academy of Medical Sciences Springboard Award (SBF006\1165) to M.M.; the European Regional Development Fund (project no. 2014-2020.4.01.15-0012 and the Center of Excellence in Genomics [EXCEGEN] framework) to L.H., P.P., and K.K.; and Estonian Research Council grants PRG1117 to K.K. and PRG377 to P.P. H.F.B. is funded by a UCB BIOPHARMA SPRL/BBSRC PhD Studentship (BB/P504725/1). We thank Immunoqure for the provision of monoclonal Abs (mAbs) for the pan-IFNα assay under an MTA to D.D. D.D. acknowledges support from the ANR (CE17001002). We thank Drs. Diego Catalan and Christopher Piper for critically reviewing the manuscript. The graphical abstract was created with BioRender.com.

AUTHOR CONTRIBUTIONS

H.F.B., L.H., and M.M. designed and performed experiments. T.C.R.M. provided patient and healthy control IgG. K.S. and M.V. assisted with experiments. D.A.I., R.A., C.W., and R.F.G. provided clinical information and expertise. D.D., P.P., and V.B. provided serum IFNα measurements. C.M. and K.K. designed experiments and conceptualized and supervised the study.

DECLARATION OF INTERESTS

K.K. and P.P. have ownership of the patent USA20190071499A1.

Received: June 17, 2022
Revised: August 28, 2022
Accepted: December 13, 2022
Published: January 17, 2023

REFERENCES

1. Wei, C., Anolik, J., Cappione, A., Zheng, B., Pugh-Bernard, A., Brooks, J., Lee, E.-H., Milner, E.C.B., and Sanz, I. (2007). A new population of cells lacking expression of CD27 represents a notable component of the B cell memory compartment in systemic lupus erythematosus. J. Immunol. *178*, 6624–6633. https://doi.org/10.4049/jimmunol.178.10.6624.
2. Jacobi, A.M., Reiter, K., Mackay, M., Aranow, C., Hiepe, F., Radbruch, A., Hansen, A., Burmester, G.R., Diamond, B., Lipsky, P.E., et al. (2008). Activated memory B cell subsets correlate with disease activity in systemic lupus erythematosus: delineation by expression of CD27, IgD, and CD95. Arthritis Rheum. *58*, 1762–1773. https://doi.org/10.1002/art.23498.
3. Jacobi, A.M., Odendahl, M., Reiter, K., Bruns, A., Burmester, G.R., Radbruch, A., Valet, G., Lipsky, P.E., and Dörner, T. (2003). Correlation between circulating CD27high plasma cells and disease activity in patients with systemic lupus erythematosus. Arthritis Rheum. *48*, 1332–1342. https://doi.org/10.1002/art.10949.
4. Capobianchi, M.,R., Uleri, E., Caglioti, C., and Dolei, A. (2015). Type I IFN family members: similarity, differences and interaction. Cytokine Growth Factor Rev. *26*, 103.
5. Jego, G., Palucka, A.K., Blanck, J.P., Chalouni, C., Pascual, V., and Banchereau, J. (2003). Plasmacytoid dendritic cells induce plasma cell differentiation through type I interferon and interleukin 6. Immunity *19*, 225–234. https://doi.org/10.1016/S1074-7613(03)00208-5.
6. Menon, M., Blair, P.A., Isenberg, D.A., and Mauri, C. (2016). A regulatory feedback between plasmacytoid dendritic cells and regulatory B cells is aberrant in systemic lupus erythematosus. Immunity *44*, 683–697. https://doi.org/10.1016/j.immuni.2016.02.012.
7. Blanco, P., Palucka, A.K., Gill, M., Pascual, V., and Banchereau, J. (2001). Induction of dendritic cell differentiation by IFN-α in systemic lupus erythematosus. Science *294*, 1540–1543. https://doi.org/10.1126/science.1064890.
8. Santini, S.M., Lapenta, C., Logozzi, M., Parlato, S., Spada, M., Di Pucchio, T., and Belardelli, F. (2000). Type I interferon as a powerful adjuvant for monocyte-derived dendritic cell development and activity in vitro and in Hu-PBL-SCID mice. J. Exp. Med. *191*, 1777–1788. https://doi.org/10.1084/jem.191.10.1777.
9. Blanco, P., Pitard, V., Viallard, J.F., Taupin, J.L., Pellegrin, J.L., and Moreau, J.F. (2005). Increase in activated CD8+ T lymphocytes expressing perforin and granzyme B correlates with disease activity in patients with systemic lupus erythematosus. Arthritis Rheum. *52*, 201–211. https://doi.org/10.1002/art.20745.
10. Yu, C.-F., Peng, W.-M., Oldenburg, J., Hoch, J., Bieber, T., Limmer, A., Hartmann, G., Barchet, W., Eis-Hübinger, A.M., and Novak, N. (2010). Human plasmacytoid dendritic cells support Th17 cell effector function in response to TLR7 ligation. J. Immunol. *184*, 1159–1167. https://doi.org/10.4049/jimmunol.0901706.
11. Le Bon, A., Durand, V., Kamphuis, E., Thompson, C., Bulfone-Paus, S., Rossmann, C., Kalinke, U., and Tough, D.F. (2006). Direct stimulation of T cells by type I IFN enhances the CD8 + T cell response during cross-priming. J. Immunol. *176*, 4682–4689. https://doi.org/10.4049/jimmunol.176.8.4682.
12. Litinskiy, M.B., Nardelli, B., Hilbert, D.M., He, B., Schaffer, A., Casali, P., Cerutti, A., and Sciences, G. (2002). DCs induce CD40-independent

immunoglobulin class switching through BLyS and APRIL. Nat. Immunol. *3*, 822–829. https://doi.org/10.1038/ni829.

13. Lood, C., Gullstrand, B., Truedsson, L., Olin, A.I., Alm, G.V., Rönnblom, L., Sturfelt, G., Eloranta, M.L., and Bengtsson, A.A. (2009). C1q inhibits immune complex-induced interferon-α production in plasmacytoid dendritic cells. Arthritis Rheum. *60*, 3081–3090. https://doi.org/10.1002/art.24852.
14. Wu, J., Wilson, J., He, J., Xiang, L., Schur, P.H., and Mountz, J.D. (1996). Fas ligand mutation in a patient with systemic lupus erythematosus and lymphoproliferative disease. J. Clin. Invest. *98*, 1107–1113. https://doi.org/10.1172/JCI118892.
15. Rice, G.I., Rodero, M.P., and Crow, Y.J. (2015). Human disease phenotypes associated with mutations in TREX1. J. Clin. Immunol. *35*, 235–243. https://doi.org/10.1007/s10875-015-0147-3.
16. Wandl, U.B., Nagel-Hiemke, M., May, D., Kreuzfelder, E., Kloke, O., Kranzhoff, M., Seeber, S., and Niederle, N. (1992). Lupus-like autoimmune disease induced by interferon therapy for myeloproliferative disorders. Clin. Immunol. Immunopathol. *65*, 70–74. https://doi.org/10.1016/0090-1229(92)90250-R.
17. Ho, V., Mclean, A., and Terry, S. (2008). Severe systemic lupus erythematosus induced by antiviral treatment for hepatitis C. J. Clin. Rheumatol. *14*, 166–168.
18. Houssiau, F.A., Thanou, A., Mazur, M., Ramiterre, E., Gomez Mora, D.A., Misterska-Skora, M., Perich-Campos, R.A., Smakotina, S.A., Cerpa Cruz, S., Louzir, B., et al. (2020). IFN-α kinoid in systemic lupus erythematosus: results from a phase IIb, randomised, placebo-controlled study. Ann. Rheum. Dis. *79*, 347–355. https://doi.org/10.1136/annrheumdis-2019-216379.
19. Furie, R.A., Morand, E.F., Bruce, I.N., Manzi, S., Kalunian, K.C., Vital, E.M., Lawrence Ford, T., Gupta, R., Hiepe, F., Santiago, M., et al. (2019). Type I interferon inhibitor anifrolumab in active systemic lupus erythematosis (TULIP-1): a randomised, controlled, phase 3 trial. Lancet Rheumatol. *1*, 208–219.
20. Chatham, W.W., Furie, R., Saxena, A., Brohawn, P., Schwetje, E., Abreu, G., and Tummala, R. (2021). Long-term safety and efficacy of anifrolumab in adults with systemic lupus erythematosus: results of a phase II open-label extension study. Arthritis Rheumatol. *73*, 816–825. https://doi.org/10.1002/art.41598.
21. van der Eijk, A.A., Vrolijk, J.M., and Haagmans, B.L. (2006). Antibodies neutralizing peginterferon alfa during retreatment of hepatitis C. N. Engl. J. Med. *354*, 1323–1324. https://doi.org/10.1056/nejmc052880.
22. Sorensen, P.S., Ross, C., Clemmesen, K.M., Bendtzen, K., Frederiksen, J.L., Jensen, K., Kristensen, O., Petersen, T., Rasmussen, S., Ravnborg, M., et al. (2003). Clinical importance of neutralising antibodies against interferon beta in patients with relapsing-remitting multiple sclerosis. Lancet *362*, 1184–1191. https://doi.org/10.1016/S0140-6736(03)14541-2.
23. Meager, A., Visvalingam, K., Peterson, P., Möll, K., Murumägi, A., Krohn, K., Eskelin, P., Perheentupa, J., Husebye, E., Kadota, Y., et al. (2006). Anti-interferon autoantibodies in autoimmune polyendocrinopathy syndrome type 1. PLoS Med. *3*, e289. https://doi.org/10.1371/journal.pmed.0030289.
24. Kisand, K., Link, M., Wolff, A.S.B., Meager, A., Tserel, L., Org, T., Murumägi, A., Uibo, R., Willcox, N., Trebusak Podkrajsek, K., et al. (2008). Interferon autoantibodies associated with AIRE deficiency decrease the expression of IFN-stimulated genes. Blood *112*, 2657–2666. https://doi.org/10.1182/blood-2008-03-144634.
25. Meager, A., Vincent, A., Newsom-Davis, J., and Willcox, N. (1997). Spontaneous neutralising antibodies to interferon- and interleukin-12 in thymoma- associated autoimmune disease. Lancet *350*, 1596–1597.
26. Gupta, S., Tatouli, I.P., Rosen, L.B., Hasni, S., Alevizos, I., Manna, Z.G., Rivera, J., Jiang, C., Siegel, R.M., Holland, S.M., et al. (2016). Distinct functions of autoantibodies against interferon in systemic lupus erythematosus: a comprehensive analysis of anticytokine autoantibodies in common rheumatic diseases. Arthritis Rheumatol. *68*, 1677–1687. https://doi.org/10.1002/art.39607.
27. Morimoto, A.M., Flesher, D.T., Yang, J., Wolslegel, K., Wang, X., Brady, A., Abbas, A.R., Quarmby, V., Wakshull, E., Richardson, B., et al. (2011). Association of endogenous anti-interferon-α autoantibodies with decreased interferon-pathway and disease activity in patients with systemic lupus erythematosus. Arthritis Rheum. *63*, 2407–2415. https://doi.org/10.1002/art.30399.
28. Mathian, A., Breillat, P., Dorgham, K., Bastard, P., Charre, C., Lhote, R., Quentric, P., Moyon, Q., Mariaggi, A.-A., Mouries-Martin, S., et al. (2022). Lower disease activity but higher risk of severe COVID-19 and herpes zoster in patients with systemic lupus erythematosus with pre-existing autoantibodies neutralising IFN-α. Ann. Rheum. Dis. *81*, 1695–1703. https://doi.org/10.1136/ard-2022-222549.
29. Bastard, P., Rosen, L.B., Zhang, Q., Michailidis, E., Hoffmann, H.H., Zhang, Y., Dorgham, K., Philippot, Q., Rosain, J., Béziat, V., et al. (2020). Autoantibodies against type I IFNs in patients with life-threatening COVID-19. Science *370*, eabd4585. https://doi.org/10.1126/science.abd4585.
30. Bastard, P., Michailidis, E., Hoffmann, H., Chbihi, M., Voyer, T., Rosain, J., Philippot, Q., Seeleuthner, Y., Gervais, A., Materna, M., et al. (2021). Autoantibodies to type I IFNs can underlie adverse reactions to yellow fever live attenuated vaccine. J. Exp. Med. *218*, e20202486.
31. Rodero, M.P., Decalf, J., Bondet, V., Hunt, D., Rice, G.I., Werneke, S., McGlasson, S.L., Alyanakian, M.A., Bader-Meunier, B., Barnerias, C., et al. (2017). Detection of interferon alpha protein reveals differential levels and cellular sources in disease. J. Exp. Med. *214*, 1547–1555. https://doi.org/10.1084/jem.20161451.
32. Meyer, S., Woodward, M., Hertel, C., Vlaicu, P., Haque, Y., Kärner, J., Macagno, A., Onuoha, S.C., Fishman, D., Peterson, H., et al. (2016). AIRE-deficient patients harbor unique high-affinity disease-ameliorating autoantibodies. Cell *166*, 582–595. https://doi.org/10.1016/j.cell.2016.06.024.
33. Kirou, K.A., Lee, C., George, S., Louca, K., Papagiannis, I.G., Peterson, M.G.E., Ly, N., Woodward, R.N., Fry, K.E., Lau, A.Y.H., et al. (2004). Coordinate overexpression of interferon-α-induced genes in systemic lupus erythematosus. Arthritis Rheum. *50*, 3958–3967. https://doi.org/10.1002/art.20798.
34. Kärner, J., Pihlap, M., Ranki, A., Krohn, K., Trebusak Podkrajsek, K., Bratanic, N., Battelino, T., Willcox, N., Peterson, P., and Kisand, K. (2016). IL-6-specific autoantibodies among APECED and thymoma patients. Immun. Inflamm. Dis. *4*, 235–243. https://doi.org/10.1002/iid3.109.
35. Finkelman, F.D., Madden, K.B., Morris, S.C., Holmes, J.M., Boiani, N., Katona, I.M., and Maliszewski, C.R. (1993). Anti-cytokine antibodies as carrier proteins. Prolongation of in vivo effects of exogenous cytokines by injection of cytokine-anti-cytokine antibody complexes. J. Immunol. *151*, 1235–1244.
36. Spangler, J.B., Tomala, J., Luca, V.C., Jude, K.M., Dong, S., Ring, A.M., Votavova, P., Pepper, M., Kovar, M., and Garcia, K.C. (2015). Antibodies to interleukin-2 elicit selective T cell subset potentiation through distinct conformational mechanisms. Immunity *42*, 815–825. https://doi.org/10.1016/j.immuni.2015.04.015.
37. Liu, M., Guo, Q., Wu, C., Sterlin, D., Goswami, S., Zhang, Y., Li, T., Bao, C., Shen, N., Fu, Q., et al. (2019). Type I interferons promote the survival and proinflammatory properties of transitional B cells in systemic lupus erythematosus patients. Cell. Mol. Immunol. *16*, 367–379. https://doi.org/10.1038/s41423-018-0010-6.
38. Bocharnikov, A.v., Keegan, J., Wacleche, V.S., Cao, Y., Fonseka, C.Y., Wang, G., Muise, E.S., Zhang, K.X., Arazi, A., Keras, G., et al. (2019). PD-1hiCXCR5- T peripheral helper cells promote B cell responses in lupus via MAF and IL-21. JCI Insight *4*, e130062. https://doi.org/10.1172/jci.insight.130062.
39. Menon, M., Blair, P.A., Isenberg, D.A., and Mauri, C. (2016). A regulatory feedback between plasmacytoid dendritic cells and regulatory B cells is

aberrant in systemic lupus erythematosus. Immunity *44*, 683–697. https://doi.org/10.1016/j.immuni.2016.02.012.

40. Wang, E.Y., Mao, T., Klein, J., Dai, Y., Huck, J.D., Liu, F., Zheng, S., Zhou, T., Israelow, B., Wong, P., et al. (2020). Diverse functional autoantibodies in patients with COVID-19. Preprint at medRxiv. https://doi.org/10.1101/2020.12.10.20247205.

41. Andersen, J.T., Dalhus, B., Viuff, D., Ravn, B.T., Gunnarsen, K.S., Plumridge, A., Bunting, K., Antunes, F., Williamson, R., Athwal, S., et al. (2014). Extending serum half-life of albumin by engineering neonatal Fc receptor (FcRn) binding. J. Biol. Chem. *289*, 13492–13502. https://doi.org/10.1074/jbc.M114.549832.

42. Rosenblum, M.G., Unger, B.W., Gutterman, J.U., Hersh, E.M., David, G.S., and Frincke, J.M. (1985). Modification of human leukocyte interferon pharmacology with a monoclonal antibody. Cancer Res. *45*, 2421–2424.

43. Hochberg, M.C. (1997). Updating the American College of Rheumatology revised criteria for the classification of systemic lupus erythematosus. Arthritis Rheum. *40*, 1725. https://doi.org/10.1002/art.1780400928.

44. Tan, E.M., Cohen, A.S., Fries, J.F., Masi, A.T., Mcshane, D.J., Rothfield, N.F., Schaller, J.G., Talal, N., and Winchester, R.J. (1982). The 1982 revised criteria for the classification of systemic lupus erythematosus. Arthritis Rheum. *25*, 1271–1277. https://doi.org/10.1002/art.1780251101.

45. Yee, C.S., Cresswell, L., Farewell, V., Rahman, A., Teh, L.S., Griffiths, B., Bruce, I.N., Ahmad, Y., Prabu, A., Akil, M., et al. (2010). Numerical scoring for the BILAG-2004 index. Rheumatology *49*, 1665–1669. https://doi.org/10.1093/rheumatology/keq026.

46. Gordon, C., Amissah-Arthur, M.B., Gayed, M., Brown, S., Bruce, I.N., D'Cruz, D., Empson, B., Griffiths, B., Jayne, D., Khamashta, M., et al. (2018). The British Society for Rheumatology guideline for the management of systemic lupus erythematosus in adults. Rheumatology *57*, e1–e45. https://doi.org/10.1093/rheumatology/kex286.

STAR★METHODS

KEY RESOURCES TABLE

REAGENT or RESOURCE	SOURCE	IDENTIFIER
**Antibodies**		
Anti-pan-IFNa (capture), 8H1 clone	ImmunoQure	N/A
Anti-pan-IFNa (detection), 12H15 clone	ImmunoQure	N/A
Biotin mouse anti-Human IgG1	BD Pharmingen	Cat# 555869; RRID:AB_396187
Biotin Mouse anti-human IgG2	BD Pharmingen	Cat# 555874; RRID:AB_396190
Biotin Mouse anti-Human IgG4	BD Pharmingen	Cat# 555882; RRID:AB_396194
Monoclonal anti-Human IgG3-Biotin	Sigma Aldrich	Cat# B3523-2ML; RRID:AB_258549
CD19 BV785, Clone HIB19	Biolegend	Cat# 363028; RRID:AB_2564257
CD24 APCeFluor780, Clone SN3 A5-2H10	ThermoFisher Scientific	Cat# 47-0247-42; RRID:AB_10735091
CD38 PerCPeFluor710, Clone HB7	ThermoFisher Scientific	Cat# 46-0388-42; RRID:AB_1834399
CD27 PE/Cy7, Clone M-T271	Biolegend	Cat# 356412; RRID:AB_2562258
IgD BV605, Clone IA6-2	Biolegend	Cat# 348232; RRID:AB_2563337
IL-10 APC, Clone JES5-16E3	BD Pharmingen	Cat# 17-7101-82; RRID:AB_469502
Blimp1 Alexa Fluor 488, Clone 646702	Biotechne	Cat# IC36081G; RRID:AB_11129439
CD3 Alexa Fluor 488, Clone UCHT1	Biolegend	Cat# 300415; RRID:AB_389310
CD4 PE/Dazzle595, Clone A161A1	Biolegend	Cat# 357412; RRID:AB_2565664
CXCR5 BV421, Clone J252D4	Biolegend	Cat# 356920; RRID:AB_2562303
CCR7 BV785, Clone G043H7	Biolegend	Cat# 353230; RRID:AB_2563630
ICOS PE/Cy7, Clone 7E.17G9	Biolegend	Cat# 117422; RRID:AB_2860637
PD-1 BUV737, Clone EH12.1	BD PharMingen	Cat# 612792; RRID:AB_2870119
**Biological samples**		
Human serum from healthy controls and SLE patients	University College London Hospital, London UK, Tartu, Estonia.	N/A
Primary human peripheral blood mononuclear cells from healthy controls and SLE patients	University College London Hospital/UCL, London, UK.	N/A
**Chemicals, peptides, and recombinant proteins**		
Recombinant human IFN-α2	Miltenyi Biotech	Cat# 130-093-874
Human IFN-Alpha Sampler Set	PBL Assay Science	Cat# 11002
Nano-Glo luciferase assay reagent	Promega	Cat# N1110
QUANTI-Blue colorimetric enzyme assay	InvivoGen	Cat# rep-gbs
CpGC ODN 2395	Invivogen	Cat# tlrl-2395
Phorbol-12-myristate-13 acetate (PMA)	Sigma Aldrich	Cat# 79346
Ionomycin	Sigma Aldrich	Cat# I9657
Brefeldin A	Sigma Aldrich	Cat# B5936
**Critical commercial assays**		
PicoPure(TM) RNA Isolation Kit	ThermoFisher Scientific	Cat# KIT0204
iScript(TM) cDNA Synthesis Kit	BioRad	Cat# 1708891
iQ(TM) SYBR(R) Green Supermix	BioRad	Cat# 1708882
RNAse-Free DNase Set	QIAGEN	Cat# 79254
Human Interferon Alpha 2 ELISA Kit	Abcam	ab233622
Multi-IFN-Alpha subtype quantification Digital ELISA kit (Simoa)	Quanterix	Beta-version
**Experimental models: Cell lines**		
HEK293	ATCC	Cat # CRL-1573; RRID:CVCL_0045
HEK-Blue IFN-α/β	InvivoGen	Cat# hkb-ifnab; RRID:CVCL_KT26

(Continued on next page)

REAGENT or RESOURCE	SOURCE	IDENTIFIER
Continued		
Oligonucleotides		
Hs_IRF9_1_SG QuantiTect Primer Assay	QIAGEN	Cat# 249900; GeneGlobe ID: QT00001113
Hs_MCL1_1_SG QuantiTect Primer Assay	QIAGEN	Cat# 249900; GeneGlobe ID: QT00094122
STAT1 Quantitect primer pair	QIAGEN	Cat# 249900; GeneGlobe ID: QT00074123
MX1 custom primer pair	ThermoFisher Scientific	Sequences provided in methods
GAPDH custom primer pair	ThermoFisher Scientific	Sequences described in methods
Recombinant DNA		
pPK-CMV-F4 fusion vector	PromoCell	N/A
Software and algorithms		
GraphPad Prism 9	Graphpad Software	http://www.graphpad.com
Flowjo v.10	Flowjo, LLC	https://flowjo.com
RStudio v 1db809b8	RStudio, PBC	https://www.rstudio.com
Other		
RPMI-1640 media	Sigma Aldrich	Cat# R8658
DMEM media	Lonza	Cat# 12-614F
Fetal calf serum (FCS)	Biosera	Cat# FB1001/500
Penicillin/Streptomycin	Sigma Aldrich	Cat# P0781
Antibiotic/Antimycotic Mix	Corning	Cat# 30-004-CI
Blasticidin	InvivoGen	Cat# anti-bl-05
Zeocin	InvivoGen	Cat# ant-zn-05
Trypsin	Corning	Cat# 25-052-CI
Lipofectamine	Invitrogen	Cat# 11668-019
OptiMem	Gibco	Cat# 31985-062
Protein G Agarose High Flow Resin	Exalpha Biologicals	Cat# COP28
Streptavidin Agarose Beads	NovaGen	Cat# 69203-3
FcR Blocking Reagent, human	Miltenyi Biotec	Cat# 130-059-901
eBioscience Intracellular Fixation and Permeabilization buffer set	ThermoFisher Scientific	Cat# 88-8824-00
LIVE/DEAD(TM) Fixable Blue Dead Cell Stain Kit	ThermoFisher Scientific	Cat# L23105
Protein G FF column (1mL)	Generon	Cat# NB-45-00,048-1-1
Amicon-15 (50kDa NWCO)	Merck	Cat# UFC505096
Pierce(TM) High Capacity Endotoxin Removal Spin Columns, 0.5mL	ThermoFisher Scientific	Cat# 88276
Pierce(TM) BCA Protein Assay Kit	ThermoFisher Scientific	Cat# 23225

RESOURCE AVAILABILITY

Lead contact

Further information and requests for resources and reagents should be directed to and will be fulfilled by the lead contact, Professor Claudia Mauri (c.mauri@ucl.ac.uk).

Materials availability

This study did not generate new unique reagents.

Data and code availability

- Data reported in this paper will be shared by the lead contact upon request.
- This paper does not report original code.
- Any additional information required to reanalyze the data reported in this work paper is available from the lead contact upon request.

EXPERIMENTAL MODEL AND SUBJECT DETAILS

Study population

Blood samples for PBMC and serum isolation were collected from SLE patients attending the University College London Hospital (UCLH) rheumatology clinic, and from healthy volunteers following informed consent. Ethical approval was obtained from the UCLH Health Service ethical committee, under REC reference no. 14/SC/1200. Sample storage complied with requirements of the Data Protection Act 1998.

The study was designed in a cross-sectional manner. During the period 2009–2020, 501 patients were recruited at University College London Hospital (UCLH) rheumatology clinic. All patients had to have a diagnosis of SLE satisfying at least 4 of the 11 American College of Rheumatology classification criteria and updated in 1997, with a disease duration of $\geq$6 months.[43,44] Patients were positive for antinuclear antibody (ANA or anti-double-stranded DNA (dsDNA) antibodies.

Exclusion criteria were an age under 18, history of treatment with rituximab, participation in any interventional trial and pregnancy. Patients with severe CNS lupus, congestive heart failure, a history of cancer, severe glomerulonephritis, a history of recurrent or active infections such as HIV, tuberculosis, hepatitis B/C viruses and a history of demyelinating disease, for example, multiple sclerosis or optic neuritis, were also excluded.

All participants underwent a structured examination by a rheumatologist. BILAG SLE criteria were recorded.[45] Disease duration was defined as the time (years) from the first point at which an SLE diagnosis was documented in the patient records, until inclusion into this cohort. Disease activity was assessed by the British Isles Lupus Assessment Group (BILAG), a standardized disease activity assessment. Blood tests are performed as part of routine clinic visits and include: anti-dsDNA (double stranded DNA) autoantibody titers, complement C3 levels, complete blood counts, urea/electrolytes/serum creatinine, leukocyturia and haematuria, and a dip stick test for protein with a protein:creatinine ratio requested if + or more is recorded. Fever was defined as a body temperature above 38.5°C, weight loss as a loss of at least 5% of body weight, and cytopenia as leukopenia <3 G/L or thrombocytopenia <100 G/L. Leukopenia related to drugs or benign ethnic causes were not scored in the BILAG.

To provide numerical scores, we used a previous weighting system that assigned a score of 9 to active manifestations (grade A in the BILAG), 3 to grade B manifestations, 1 to grade C manifestations, and 0 to grade D and E manifestations. We used the sum of these scores as a summary index (possible range 0–72).[45] Low lupus disease activity was defined as a BILAG global score of $\leq$5 with no activity in major organ systems and no hemolytic anemia or gastrointestinal activity, without new lupus disease activity compared with the previous assessment, and with corticosteroid treatment up to 7.5 mg/day of prednisone (treatment with an immunosuppressant and/or hydroxychloroquine (HCQ) were allowed).[46] In the case of multiple serum samples at different dates for the same patient, only the oldest one was included and established as day 0. The kinetics of anti-IFN-α-autoantibody levels over time were determined in all the available serum samples of patients who tested positive for anti-IFN-Ab more than once.

Demographics, clinical characteristics, routine laboratory testing and therapeutic regimen (reported in Tables 1, S2 and S3) were collected from electronical medical files of the visit to the clinic recorded on the day blood was drawn (Day 0). Healthy controls from UCLH and UCL were enrolled after informed consent.

Cell and cell lines

Primary cells

Prior to experiments, PBMCs from healthy controls and SLE patients were stored in liquid nitrogen in cryovials containing 10% DMSO and 90% fetal calf serum (FCS) and were thawed in warm RPMI 1640 (Sigma-Aldrich) supplemented with 10% FCS and 100 IU/mg penicillin/streptomycin (Sigma-Aldrich). For primary cell cultures, PBMCs were seeded in 96-well plates at a density of 5 × 10^6 cells/mL in RPMI 1640 supplemented with 10% FCS and 100 IU/mg penicillin/streptomycin. PBMCs were stimulated with 1μM CpGC ODN 2395 (InvivoGen), then incubated for 72 hrs at 37°C and 5% CO_2. For IgG cultures, PBMCs from healthy donors were cultured at 5 × 10^6 cells/mL with 1μM CpGC, sodium azide-free Fc blocking reagent (Miltenyi) and 200 μg/mL IgG isolated from healthy donors or SLE patients.

Cell lines

HEK293 cells were thawed and plated in DMEM (Lonza) containing 10% FCS and Antibiotic/Antimycotic mix (Corning) into 10mL tissue culture plates. Cells were incubated at 37°C and 5% CO_2 for 72h. Cells were washed with PBS and detached with warm trypsin (Corning) for 1 min. Trypsin was inactivated with the medium and cells pelleted, then seeded at 250,000 cells per 3mL well of a 6 well plate in DMEM containing 10% FCS and Antibiotic/Antimycotic mix. Following overnight incubation at 37°C 5% CO_2 cells were transfected with 4μg DNA, 8μL lipofectamine (Invitrogen) and 250μL OptiMem reduced serum media (Gibco).

HEK-Blue cells were thawed and plated in DMEM containing 10% heat-inactivated FCS and Antibiotic/Antimycotic mix in 10mL culture plates. Cells were incubated at 37°C and 5% CO_2 and maintained and subcultured in growth medium supplemented with 30 μg/mL blasticidin and 100 μg/mL zeocin (Invitrogen). Cells were passaged upon reaching a 70–80% confluency.

METHOD DETAILS

PBMC and serum isolation

A total of 50mL whole peripheral blood was collected from an individual patient or healthy donor for PBMC isolation using Ficoll-based density gradient centrifugation. A total of 10mL whole peripheral blood was collected into serum-separator (SST) tubes, centrifuged for 10 min at 1200 g at RT and serum decanted.

Luciferase immunoprecipitation system (LIPS) assay

LIPS was performed as previously described.[32] Briefly, different IFNα subtype and cytokine sequences were cloned into modified pPK-CMV-F4 fusion vector (PromoCell GmbH, Germany) where Firefly luciferase was substituted in the plasmid for *NanoLuc* luciferase (Promega, USA). Cloned constructs were transfected into HEK293 cells (ATCC), and after 48h tissue culture media containing fusion proteins were collected and stored at −20 °CC. IgG from the serum samples was captured onto Protein G Agarose beads (Exalpha Biologicals, USA) at room temperature for 1h in 96-well microfilter plate (Merck Millipore, Germany). Antigens were added to microfilter plate at 1×10^6 luminescence units (LU) per well and incubated at room temperature for 1h. After washing the plate with vacuum system, Nano-Glo Luciferase Assay Reagent was added (Promega, USA). Luminescence intensity was measured by VICTOR X Multilabel Plate Reader (PerkinElmer Life Sciences, USA). The results were expressed as arbitrary units (AU) representing the percent of signal intensity from a positive control sample. Positve negative discrimination level was calculated as mean plus 3 SD from 1% trimmed values of healthy controls.

For the detection of IgG subclass-specificity, serum samples were incubated with fusion protein solutions (10^6 LU per well) overnight at +4 °C. Next day, agarose beads bound with streptavidin (Novagen, USA) were incubated with biotin-conjugated human subclass-specific antibodies (anti-IgG_1, anti-IgG_2, anti-IgG_4 from BD Pharmingen, USA; anti-IgG_3 from Sigma-Aldrich, USA) in microfilter plates for 1 h at room temperature. Overnight incubated serum samples with fusion protein solutions were added to microfilter plate and incubated at room temperature for 2h. Microfilter plates were washed, and luminescence intensity measured as above. The results were expressed as luminescence units (LU).

Neutralization assay

Type I interferon neutralizing capacity was measured by using a reporter cell line HEK-Blue IFN-α/β (InvivoGen, USA) as previously described.[32] The cells were grown in DMEM (Lonza, Switzerland) with heat-inactivated 10% FBS, 30 g/mL Blasticidin (InvivoGen, USA) and 100 g/mL Zeocin (InvivoGen, USA). IFN-α2 was used at concentration 25 U/mL (Miltenyi Biotech, Germany). Serial dilutions were made to find the optimal dilution for other IFN-α subtypes (IFN-α1, IFN-α4, IFN-α5, IFN-α6, IFN-α7, IFN-α8, IFN-α10, IFN-α14, IFN-α16, IFN-α17, IFN-α21 from PBL Assay Science, USA). The dilution that induced approximately the same alkaline phosphatase (AP) concentration as IFN-α2 25 U/mL was selected for further neutralization assays.

3-fold serially diluted serum samples were co-incubated with interferons for 2h at 37 °C, 5% CO_2. 10^5 IFN-α-HEK-Blue cells were added to microtiter plate wells and incubated 20-24h at 37 °C, 5% CO_2. QUANTI-Blue (InvivoGen, USA) colorimetric enzyme assay was used to determine AP activity in overnight supernatants. Optical density (OD) was measured at 620 nm with Multiscan MCC/340 ELISA reader (Labsystems, USA). Neutralization activity was expressed as IC50, which was calculated from the dose-response curves and represents the serum dilution at which the IFN bioactivity was reduced to half of its maximum.

IFNα concentration measurement

Simoa digital ELISA was performed to measure IFNα concentration in patient serum. Patient samples were measured either with a homebrew assay previously described[31] with the specific assay details below, or with a prototype multi-IFN-α subtype assay (Quanterix) that utilises the same mAbs. Two IFNα specific antibodies (cloned from APECED patients) described previously[32] were used. The 8H1 antibody clone was used as a capture antibody after coating on paramagnetic beads (0.3 mg/mL), and the 12H5 was biotinylated (biotin/antibody ratio = 30/1) and used as the detector. The results are expressed in fg/mL, with a detection limit of 0.7 fg/mL.

IFNα concentrations in cell culture supernatants were quantified using a human interferon alpha 2 ELISA Kit (Abcam).

RT-qPCR

Total RNA was extracted from total PBMCs using the Arcturus PicoPure kit (ThermoFisher) and RNase-Free DNase Set (Qiagen) as per manufacturer's instructions. RNA was reverse transcribed to cDNA using the iScript cDNA synthesis kit (Bio-Rad) RT-qPCR was performed on cDNA samples using the iQ SYBR Green Supermix kit (Bio-Rad) according to manufacturer's instructions. PCR primers used are as follows; *MCL1*, *IRF9*, *STAT1* (QIAGEN), *MX1* (forward, 5′ CACCATTCCAAGGAGGTGCACG, reverse, 5′ AGTTTCAGCAC CAGCGGGGCA) and GAPDH (forward, 5′ CGCTCTCTGCTCCTCCTGTT, reverse 5′ GCAAATGAGCCCCAGCCTTCTC). For interferon scores, ISG relative expression values were summed and score calculated as the number of standard deviations (of summed values from healthy donors; SD(HD)) above the mean of summed healthy donor values; MEAN(HD). Cut-off values were calculated as the MEAN(HD) + 2SD(HD).

$$Interferon\ score = \frac{\{(SUM(ISIG\ rel.xp)) - MEAN\ (HD)\}}{SD\ (HD)}$$

Flow Cytometry staining and analysis

PBMCs were stained at a maximal concentration of 5×10^6 cells/mL in staining buffer in the dark (PBS, 2% FCS, 1mM EDTA) or as indicated below. For exclusion of dead cells from analysis, cells were incubated in the dark for 20 min with 1:500 Live/Dead Fixable Blue Dead Cell Stain Kit (ThermoFisher) at room temperature. Cell surface markers were stained with the following directly conjugated antibodies from BioLegend: CD19 BV785 (HIB19), IgD BV605 (IA6-2), CD27 PE/Dazzle 594 (M-T271). CD24 APCeFluor780 (SN3 A5-2H10) and CD38 PerCPeFluor710 (HB7) were purchased from eBioscience. T_{FH} staining was performed using the following directly conjugated antibodies; CD3 Alexa Fluor 488 (BioLegend, UCHT1), CD4 PE/Dazzle594 (BioLegend, A161A1), CXCR5 BV421 (BioLegend, J252D4), CCR7 BV785 (BioLegend, G043H7), ICOS PE/Cy7 (BioLegend, 2D3), and PD-1 BUV737 (BD, EH12.1). For multi-colour flow cytometric surface marker analysis cells were stained for 30 min in the dark at 4°C. Cells were incubated for 10 min at 4°C in fixation buffer containing formaldehyde (eBioscience).

For intracellular cytokine staining of cultured cells, cells were stimulated with PMA (50 ng/mL), ionomycin (250 ng/mL) and brefeldin A (5 mg/mL) for the final 5 h of culture. Surface markers and dead cells were stained as previously described. Following fixation cells were permeabilised (eBioscience) and incubated with IL-10 APC (BD, JES5-16E3), Blimp1 Alexa Fluor 488 (R&D systems, 646,702) for 40 min in the dark at 4°C. Cells were acquired using a Digital LSR II flow cytometer (Becton Dickinson).

IgG isolation from plasma

IgG was purified by affinity chromatography on an AKTA Start (GE Healthcare) using a Protein G column. Protein G column (1mL, Generon) was washed with 5 column volumes (CV) of elution buffer (0.1M Glycine, pH 2.3) before equilibration with 5 CV of binding buffer (100mM sodium phosphate, 140mM sodium chloride, pH 7.2. Plasma (500μL) was thawed at room temperature and diluted 1:1 with binding buffer and injected into a 1mL loop. Protein was injected manually before washing with 5 CV binding buffer and elution across 5mL elution buffer by isocratic wash. Eluted protein was neutralized using 100μL of Tris per 1mL elution buffer. Samples were then dialyzed using a 50kDa cut-off centrifugal concentrator (Millipore). Samples were centrifuged at 3500xg for 12 min before addition of endotoxin-free PBS to a total of 5mL twice. Sample were then quantified by BCA (Pierce) and endotoxin removed by a column method (Pierce high-capacity endotoxin removal columns). Columns were regenerated with 0.2M sodium hydroxide in 95% ethanol for 1 h at room temperature (5CV), then washed with 2M sodium chloride followed by endotoxin-free water (5CV). Columns were equilibrated with endotoxin-free PBS (5CV) and samples applied. Protein was eluted using endotoxin-free PBS and aliquoted to quantify remaining IgG using the Nanodrop system (Pierce).

QUANTIFICATION AND STATISTICAL ANALYSIS

Flow cytometric data were analyzed with FlowJo software v.10.4.1 (TreeStar). Statistical analysis was performed with GraphPad Prism (La Jolla, USA), using unpaired t tests or non-parametric analysis using the Kruskal-Wallis test with Dunn's multiple comparison test for multiple comparisons, or one-way ANOVA for data passing Shapiro-Wilk normality tests. Correlations were assessed with non-parametric Spearman correlation coefficient. A p value of <0.05 was considered as significant. ns: not significant, *$p < 0.05$, **$p < 0.01$, ***$p < 0.001$, ****$p < 0.0001$.

Cell Reports Medicine, Volume *4*

Supplemental information

Inactive disease in patients with lupus is linked to autoantibodies to type I interferons that normalize blood IFNα and B cell subsets

Hannah F. Bradford, Liis Haljasmägi, Madhvi Menon, Thomas C.R. McDonnell, Karita Särekannu, Martti Vanker, Pärt Peterson, Chris Wincup, Rym Abida, Raquel Fernandez Gonzalez, Vincent Bondet, Darragh Duffy, David A. Isenberg, Kai Kisand, and Claudia Mauri

Supplementary Figures

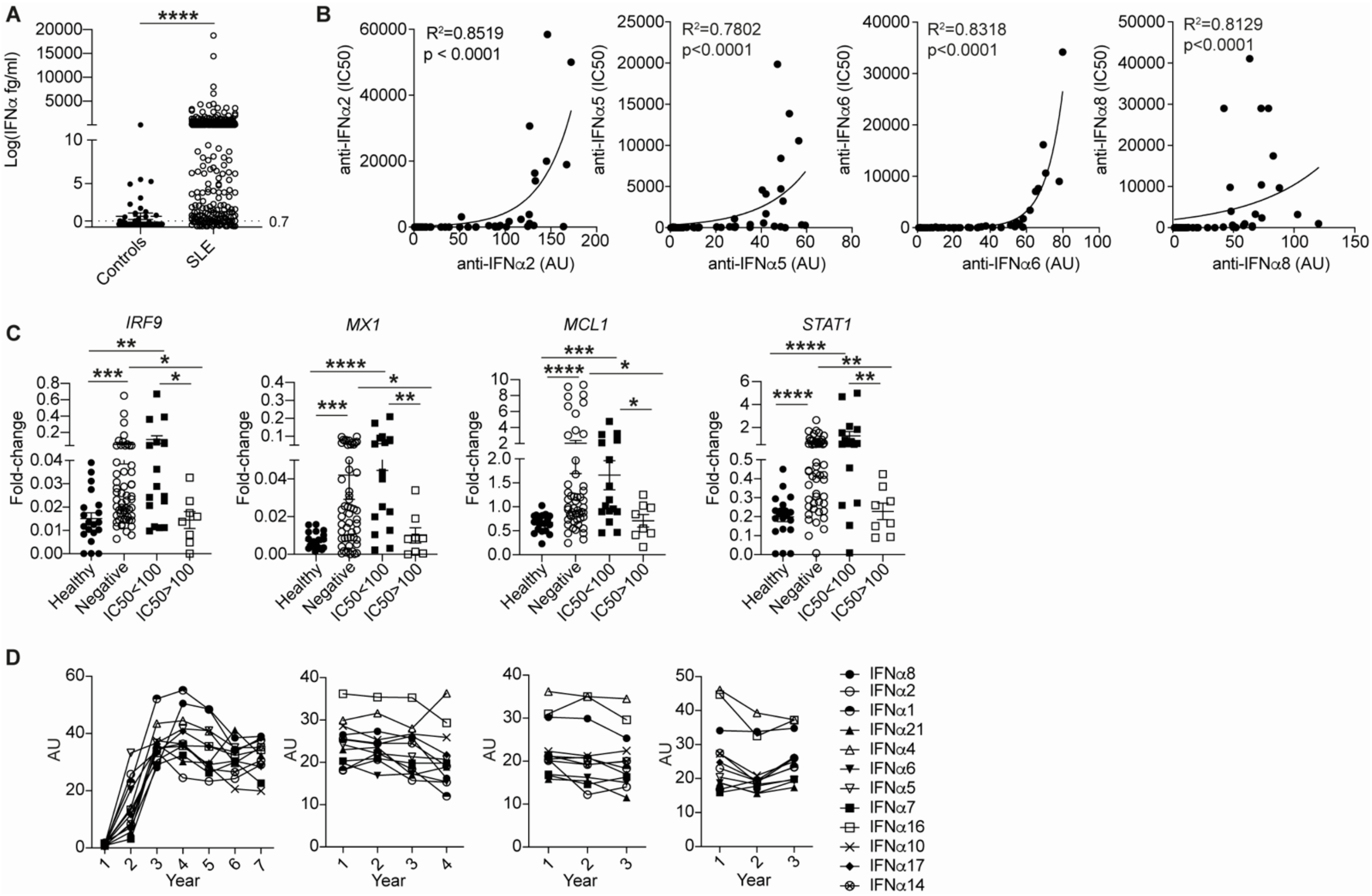

Supplementary Figure 1. High titres of anti-IFNα-autoantibodies are associated with high IFNα neutralizing capacity, reduced ISG expression and are specific to all IFNα subtypes tested (Related to Figure 1).

(A) Serum IFNα levels (fg/ml) for 54 healthy controls and 470 SLE patients.

(B) Correlations between anti-IFNα-autoantibody titres and neutralizing capacity (IC50) for autoantibody subtypes IFNα2, IFNα5, IFNα6 and IFNα8.

(C) Fold-change expression of individual ISGs *IRF9*, *MX1*, *MCL1* and *STAT1* for PBMCs isolated from SLE patients with neutralizing (n=8) or non-neutralizing anti-IFNα-autoantibodies (n=16), anti-IFNα-autoantibody negative SLE patients (n=54) and healthy controls (n=17), as measured by RT-qPCR relative to *GAPDH*.

(D) Titres of neutralizing anti-IFNα-autoantibodies against IFNα subtypes longitudinally in 4 SLE patients. *P<0.05, **P<0.01, ***P<0.001, ****P<0.0001 by unpaired Student's t-test with Welch's correction (A), two-tailed nonparametric Spearman correlation (B), Mann-Whitney test (C) or non-parametric Kruskal-Wallis test with Dunn's multiple comparison (D). Error bars are shown as mean±SEM.

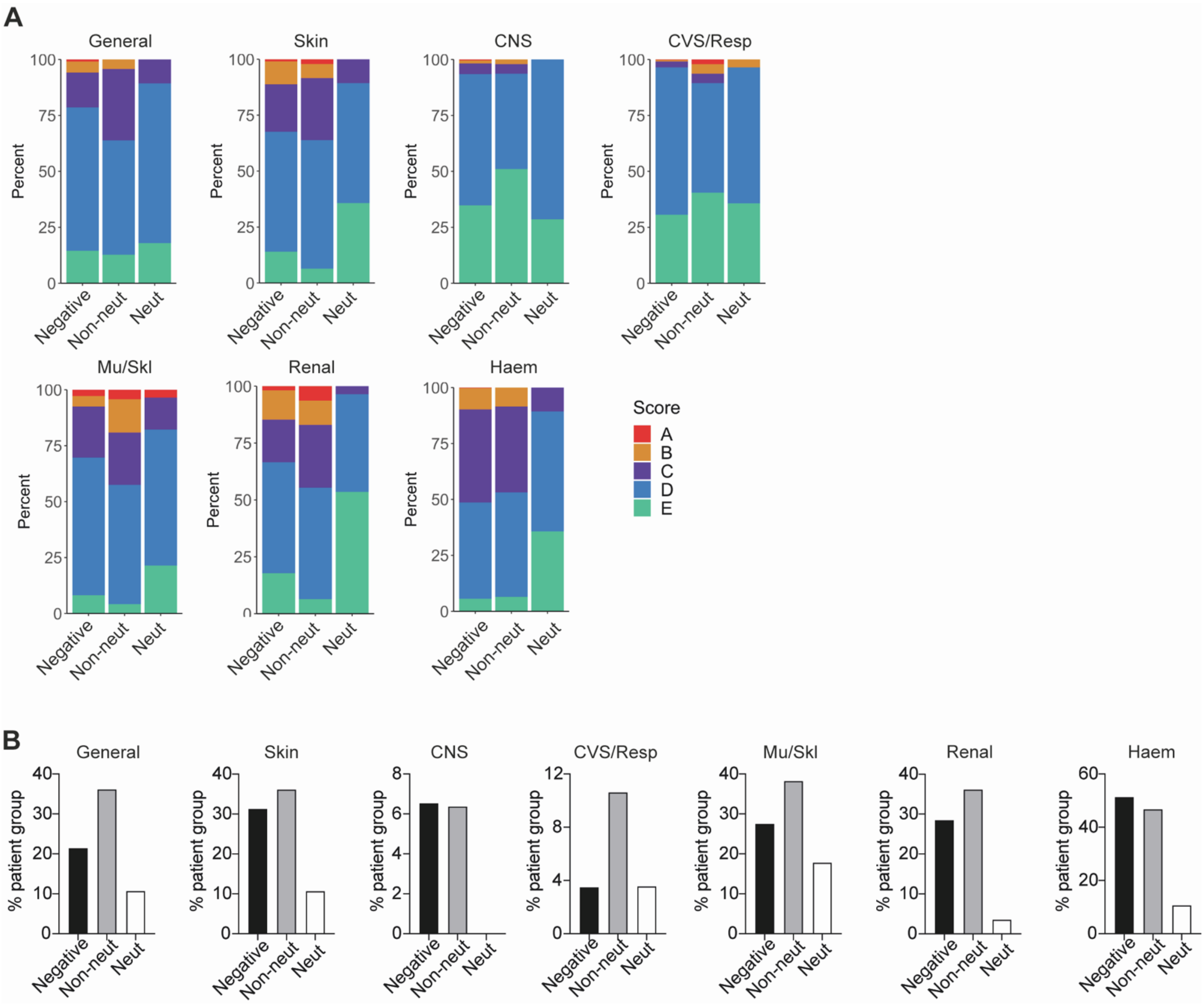

Supplementary Figure 2. Organ system involvement of SLE patients with neutralizing or non-neutralizing anti-IFNα-autoantibodies, and SLE patients lacking anti-IFNα-autoantibodies (Related to Figure 2).

(A) Percentages of SLE patients with neutralizing anti-IFNα-autoantibodies, non-neutralizing anti-IFNα-autoantibodies or anti-IFNα-autoantibody negative patients with A, B, C, D or E BILAG scores within each organ system.

(B) Percentages of SLE patients with high disease activity (a BILAG score of A, B or C) in individual organ systems, within patients with neutralizing or non-neutralizing anti-IFNα-autoantibodies, or anti-IFNα-autoantibody negative patients. Abbreviations: central nervous system (CNS), cardiovascular/respiratory (CVS/Resp), musculoskeletal (Mu/Skl), haematological (Haem).

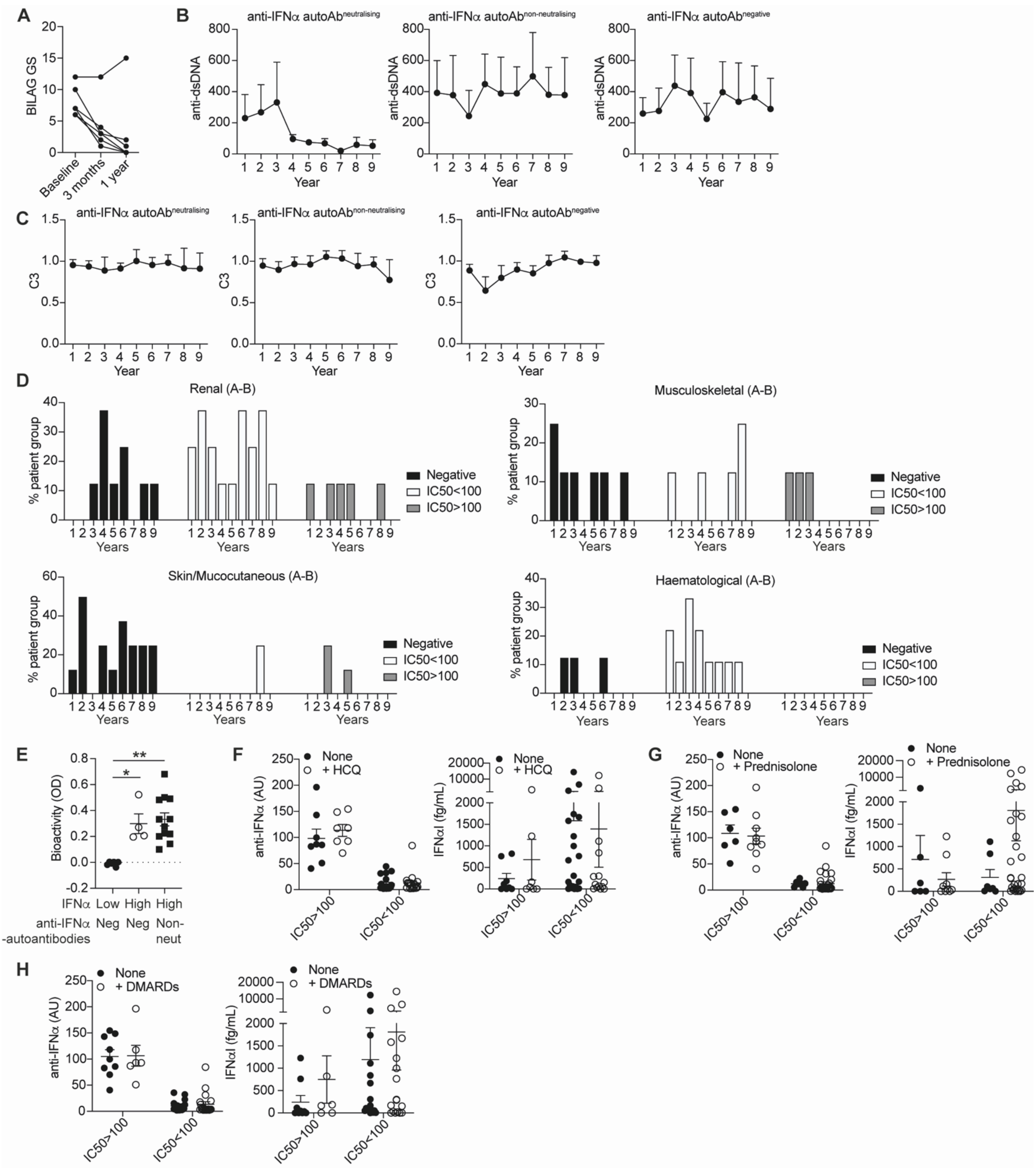

Supplementary Figure 3. Longitudinal association of clinical laboratory parameters and organ involvement for patients with neutralizing, non-neutralizing or negative anti-IFNα-autoantibodies, and cross-sectional association of treatment regime with anti-IFNα-autoantibody titres and serum IFNα levels (Related to Figure 2).

(A) Graph shows BILAG global scores (GS) for 6 patients with high disease activity at the time of initial sampling (baseline) and at 4 subsequent time points over a period of 3 years.

(B-C) Longitudinal analysis of serum (B) anti-dsDNA autoantibody titres and (C) C3 levels for SLE patients with neutralizing anti-IFNα-autoantibodies (n=11), non-neutralizing anti-IFNα-autoantibodies (n=10) and anti-IFNα-autoantibody negative patients (n=9).

(D) Graphs show percentages of patient groups with high disease activity (BILAG score of A or B) in renal, musculoskeletal, skin and haematological organ systems, for patients studied longitudinally with neutralizing anti-IFNα-autoantibodies (n=11), non-neutralizing anti-IFNα-autoantibodies (n=10) or anti-IFNα-autoantibody negative patients (n=9).

(E) Graph shows the bioactivity (OD) of IFNα in the sera from anti-IFNα-autoantibody negative patients with low or high levels of circulating IFNα, and from patients with non-neutralizing anti-IFNα-autoantibodies with high levels of circulating IFNα.

(F-H) Graphs show anti-IFNα-autoantibody titres and serum IFNα concentrations for SLE patients with neutralizing (IC50>100) versus non-neutralizing (IC50<100) anti-IFNα-autoantibodies who are receiving **(**F) hydroxychloroquine (HCQ), (G) prednisolone or (H) disease-modifying anti-rheumatic drugs (DMARDs, including methotrexate, mycophenolate mofetil and azathioprine).

$^{*}P<0.05$, $^{**}P<0.01$ by Kruskal-Wallis test with Dunn’s multiple comparison. Error bars are shown as mean±SEM.

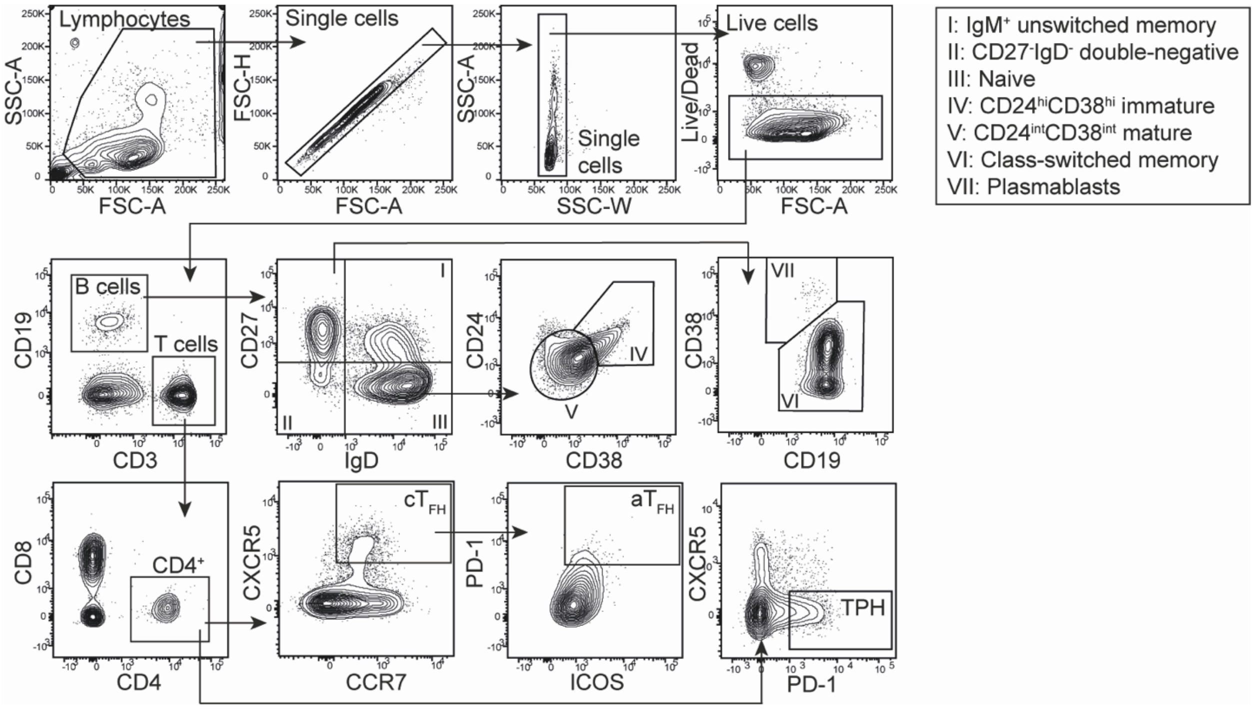

Supplementary Figure 4. Gating strategy for *ex vivo* measurement of B cell subset and T_{FH}/TPH subset frequencies by flow cytometry (Related to Figure 3 and Supplementary Figure 5).

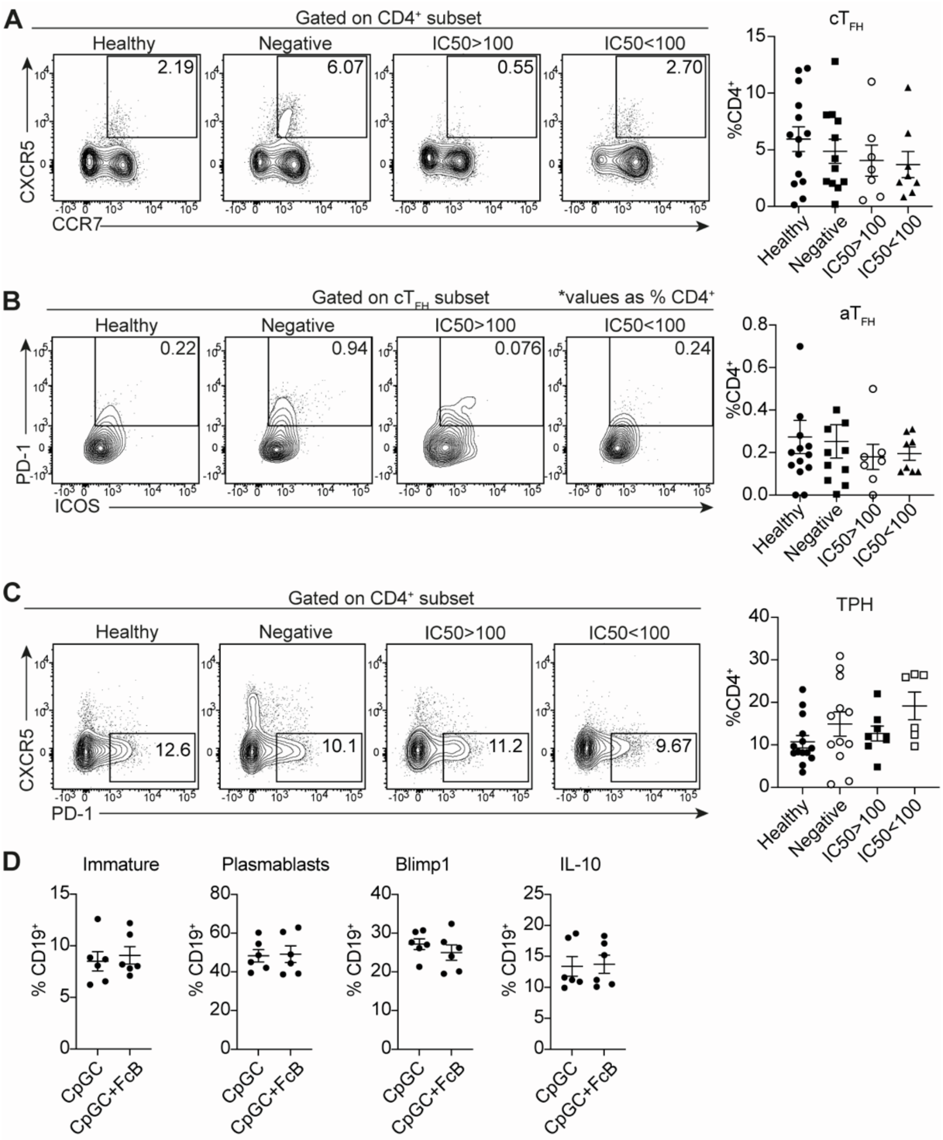

Supplementary Figure 5. Frequencies of circulating T follicular and T peripheral helper cells in SLE patients with neutralizing, non-neutralizing and negative anti-IFNα-autoantibodies and in healthy controls (Related to Figure 3).

(A-C) Representative contour plots and graphs show *ex vivo* frequencies of (A) classical T_{FH} (cT_{FH}), (B) activated T_{FH} (aT_{FH}) T cells and (C) T peripheral helper cells (TPH) in SLE patients with neutralizing anti-IFNα-autoantibodies (n=7), non-neutralizing anti-IFNα-autoantibodies (n=8), anti-IFNα-autoantibody negative patients (n=12 for cT_{FH}, n=11 for aT_{FH}) and healthy donors (n=14). Values are given as % total $CD4^+$ population.

(D) Graphs show frequencies of immature B cells, plasmablasts, $Blimp1^+$ and $IL\text{-}10^+$ B cells within the $CD19^+$ population following 72h stimulation of PBMCs with CpGC with and without Fc blocking reagent (FcB).

Supplementary Table 1. Prevalence of anti-cytokine autoantibodies in SLE patients and healthy controls (Related to Figure 1).

Seroactivity	Patients (%,n) (n=474)	Controls (%,n) (n=312)	Fisher's exact test:
IFN-α pool	14 (66)	2.2 (7)	$P < 0.0001$
IFN-ω	12 (59)	1.0 (3)	$P < 0.0001$
IFN-λ pool	2 (8)	1.0 (3)	$P = 0.5404$
IFN-β	1 (5)	0 (0)	$P = 0.1631$
IFN-γ	4 (18)	1.9 (6)	$P = 0.2025$
Th17 pool	6 (28)	1.6 (5)	$P = 0.0031$
IL pool	6 (28)	0.3 (1)	$P < 0.0001$
TNF pool	7 (35)	0.3 (1)	$P < 0.0001$
IL-1 pool	1 (4)	2.9 (9)	$P = 0.0421$
None	47 (223)	88.8 (277)	$P < 0.0001$

474 SLE patients and 312 healthy controls tested for autoantibodies against various cytokines with the Luciferase Immunoprecipitation system (LIPS) assay. The autoantibodies to cytokines were measured in groups that included an IFNα pool (IFNα1, IFNα2, IFNα8, IFNα21), IFNω, IFNγ, IFNβ1, a T helper (Th)17 pool, (IL-17A, IL-17F, IL-22), an IFNλ pool (IL-28A, IL-28B, IL-29), an interleukin (IL) pool (IL-6, IL-7, IL-10, IL-15), and a tumor necrosis factor (TNF) pool (TNF, LTA, BAFF, APRIL).

Supplementary Table 2. Demographic characteristics of SLE patients analysed longitudinally (Related to Figure 2).

Anti-IFNa autoAb	Neutralizing (IC50>100) (n=11)	Non-neutralizing (IC50<100) (n=10)	Negative (n=9)
Average age at start (years)	40.9 (24-67)	37.5 (17-70)	41.4 (21-61)
Average disease duration at start (years)	12.5 (3-25)	8.74 (0.6-20.0)	7.4 (1-15.2)
Ethnicity C/AC/SA/EA (%)	45.5/36.4/9.1/9.1	30.0/50.0/10.0/10.0	44.4/33.3/0.0/22.2
Gender F/M (%)	87.5/12.5	90.0/10.0	100.0/0.0

Abbreviations: Caucasian (C), Afro-Caribbean (A/C), South Asian (SA), East Asian (EA), Female (F), Male (M).

Supplementary Table 3. Demographics of SLE patients and healthy controls used for *ex vivo* B cell phenotyping. (Related to Figure 3).

	Healthy (n=15)	Negative (n=40)	Non-neutralizing (n=13)	Neutralizing (n=10)
Age (range)	38.2 (24-60)	47.38 (26-83)	43.54 (30-75)	45.6 (29-67)
Gender (%F:M)	86.7:13.3	97.5:2.5	84.6:23.1	100:0
Ethnicity % (C/AC/SA/EA)	53.3/13.3/13.3 /20	35/42.5/10/12.5	30.8/38.5/7.7/23.1	30/40/10/10
GS (Avg)	-	4.25	5.6	1.9
dsDNA (Avg)	-	91.4	103.1	70.1
C3 (Avg)	-	1	0.97	1.06
Organ involvement % (A/B/C/D/E)				
General	-	0/0/10/75/15	0/0/7.7/84.6/7.7	0/0/10/90/0
Skin	-	0/5/22.5/60/12.5	0/7.7/15.4/76.9/0	0/0/20/80/0
CNS	-	0/0/5/55/40	0/0/7.7/84.6/7.7	0/0/0/100/0
CVS/Resp	-	0/2.5/0/62.5/32.5	0/0/0/84.6/15.4	0/0/0/100/0
Mu/Skl	-	0/7.5/5/70/17.5	7.7/0/7.7/84.6/0	0/0/0/20/80
Renal	-	0/20/15/50/15	7.7/23.1/30.8/38.5/0	0/10/30/60/0
Haem	-	0/2.5/35/52.5/10	0/0/53.8/46.2/0	0/0/70/30/0

Abbreviations: Caucasian (C), Afro/Caribbean (A/C), South Asian (SA), East Asian (EA), global score (GS), double-stranded DNA (dsDNA), central nervous system (CNS), cardiovascular/respiratory (CVS/Resp), musculoskeletal (Mu/Skl), haematological (Haem).
